# A Multicellular Coordinated Network Driving Lymphovascular Space Invasion in Endometrioid Endometrial Carcinoma

**DOI:** 10.1111/cpr.70246

**Published:** 2026-06-23

**Authors:** Wendi Guo, Tianxiang Liu, Runling Ren, Na Li, Wenwen Zhang, Jingwen Si, Yongjun Piao, Yuanjing Hu

**Affiliations:** ^1^ Tianjin Central Hospital of Gynecology Obstetrics, School of Medicine Nankai University Tianjin China; ^2^ School of Medicine Nankai University Tianjin China; ^3^ Tianjin Central Hospital of Gynecology Obstetrics Tianjin China; ^4^ Tianjin Institute of Gynecology Obstetrics Tianjin Central Hospital of Gynecology Obstetrics Tianjin China; ^5^ Department of Pathology Tianjin Central Hospital of Gynecology Obstetrics Tianjin China

## Abstract

Lymphovascular space invasion (LVSI) is a critical factor linked to metastasis and poor outcomes in endometrioid endometrial carcinoma (EEC), yet its multicellular mechanisms remain unclear. Using single‐cell RNA sequencing of 3 LVSI‐present (LVSI+) EECs, 2 LVSI‐absent (LVSI−) EECs, and 2 normal endometrial samples, we delineated the cellular ecosystems underlying LVSI. LVSI+ EECs exhibited marked epithelial reprogramming, transitioning from differentiated ciliated epithelium to hyperproliferative and metabolically remodelled phenotypes, and contained TC4, a metastatic epithelial subset characterized by hypoxia, partial epithelial–mesenchymal transition, immunosuppression, and progesterone resistance. We also identified nine key genes in malignant epithelial cells associated with LVSI prognosis. The tumour microenvironment in LVSI+ EECs shifted from an inflammatory state dominated by epithelial cells to a collaborative network involving stromal and immune cells. This network was enriched in immunosuppressive ZNF683 + SOX4 + CD8+ T cells, Cycling_T cells, SPP1 + MMP9 + Mac, WNT5A_mCAF, Tip endothelial cells, and lymphatic endothelial cells. WNT5A_mCAF and SPP1 + MMP9 + Mac synergistically remodelled the extracellular matrix, promoted lymphangiogenesis and angiogenesis, and, together with endothelial cells, suppressed T‐cell activity via LGALS9–HAVCR2/CD44 inhibitory signalling, thereby establishing a niche conducive to vascular invasion. Using spatial multiplex immunofluorescence, we confirmed hypoxic tumour epithelial cells at the invasive front coexisting with an immunosuppressive microenvironment, and revealed spatial colocalization of PD‐L1+ tumour cells, PD‐L1+ macrophages, and PD‐1+ T cells within LVSI thrombi. Our comprehensive study provides deeper insights into LVSI as an actively coordinated multicellular process, potentially improving LVSI risk prediction, supporting treatment decision‐making, and informing new therapies targeting the tumour microenvironment.

## Graphical abstract

Single‐cell and spatial profiling reveal that lymphovascular space invasion in endometrioid endometrial carcinoma is driven by coordinated interactions among metastatic epithelium, fibroblasts, immune cells, and endothelium. This multicellular network promotes matrix remodelling, angiogenesis, and immune evasion, forming a pro‐metastatic niche that informs risk prediction, fertility‐sparing decision‐making, and microenvironment‐targeted therapies.

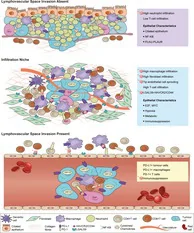

## 1 Introduction

Endometrial cancer (EC) is the most common gynecologic malignancy in developed countries, with increasing incidence and mortality rates [1]. The demand for uterine preservation is increasing among patients, yet standard hysterectomy precludes this option. Current ESGO/ESHRE/ESGE guidelines recommend conservative treatment only for a narrow subset of low‐risk patients: those with grade 1 endometrioid endometrial carcinoma (EEC) at FIGO 2009 stage IA without myometrial invasion. This limited indication highlights the challenge of safely expanding treatment options while maintaining oncologic safety.

Clinical evidence suggests that grade 1 EEC with superficial myometrial invasion is not an absolute contraindication for conservative treatment [2]. However, a key challenge is that myometrial invasion is a high‐risk factor for lymphovascular space invasion (LVSI) [3, 4], which is a strong predictor of recurrence and lymph node metastasis [5, 6, 7]. Therefore, a major clinical challenge is to develop methods for precise LVSI risk assessment in patients with some myometrial invasion to safely expand the indications for individualized treatment strategies.

LVSI, the presence of tumour cells in lymphatic or vascular channels, is the earliest pathological event in metastasis [8]. It is a key independent prognostic factor in EEC [9, 10, 11], incorporated into the 2023 FIGO staging system [12, 13], and retains prognostic value even with molecular classification [14]. Understanding its formation is critical for risk stratification. Single‐cell RNA sequencing (scRNA‐seq) allows for detailed analysis of the tumour metastasis microenvironment (TMEM) [8, 15, 16, 17, 18], a key driver of tumour progression [19]. However, the specific cell subsets and communication networks that mediate LVSI in EEC remain largely unknown [20, 21].

To investigate the cellular and molecular basis of LVSI, we performed scRNA‐seq on seven samples: 3 LVSI‐present (LVSI+) EECs, 2 LVSI‐absent (LVSI−) EECs, and 2 normal endometria. By combining a single‐cell atlas with in situ validation, our study identifies crucial cell subsets involved in LVSI and reveals alterations in tumour characteristics, the immune environment, and cell–cell communication networks. These findings provide new insights into LVSI mechanisms and offer a biological basis for LVSI risk prediction and the expansion of more tailored uterine‐preserving treatment strategies.

## 2 Results

### 2.1 Cellular Landscapes of LVSI+ EEC Revealed by scRNA‐Seq Analysis

To understand the cellular composition in LVSI+ EEC, we performed scRNA‐seq on endometrial tissues from seven donors (Figure 1A), including 3 LVSI+ EEC (two LVSI‐local, one LVSI‐substantial), 2 LVSI− EEC, and 2 normal tissues (Figure S1A). After quality control (Figure S1B), we obtained single‐cell transcriptomes for 67,062 cells with 30,421 genes, comprising 22,434 cells from LVSI+ EECs, 17,433 from LVSI− EECs, and 27,195 from normal tissues (Figure 1B; Figure S1C,D).

**FIGURE 1:**
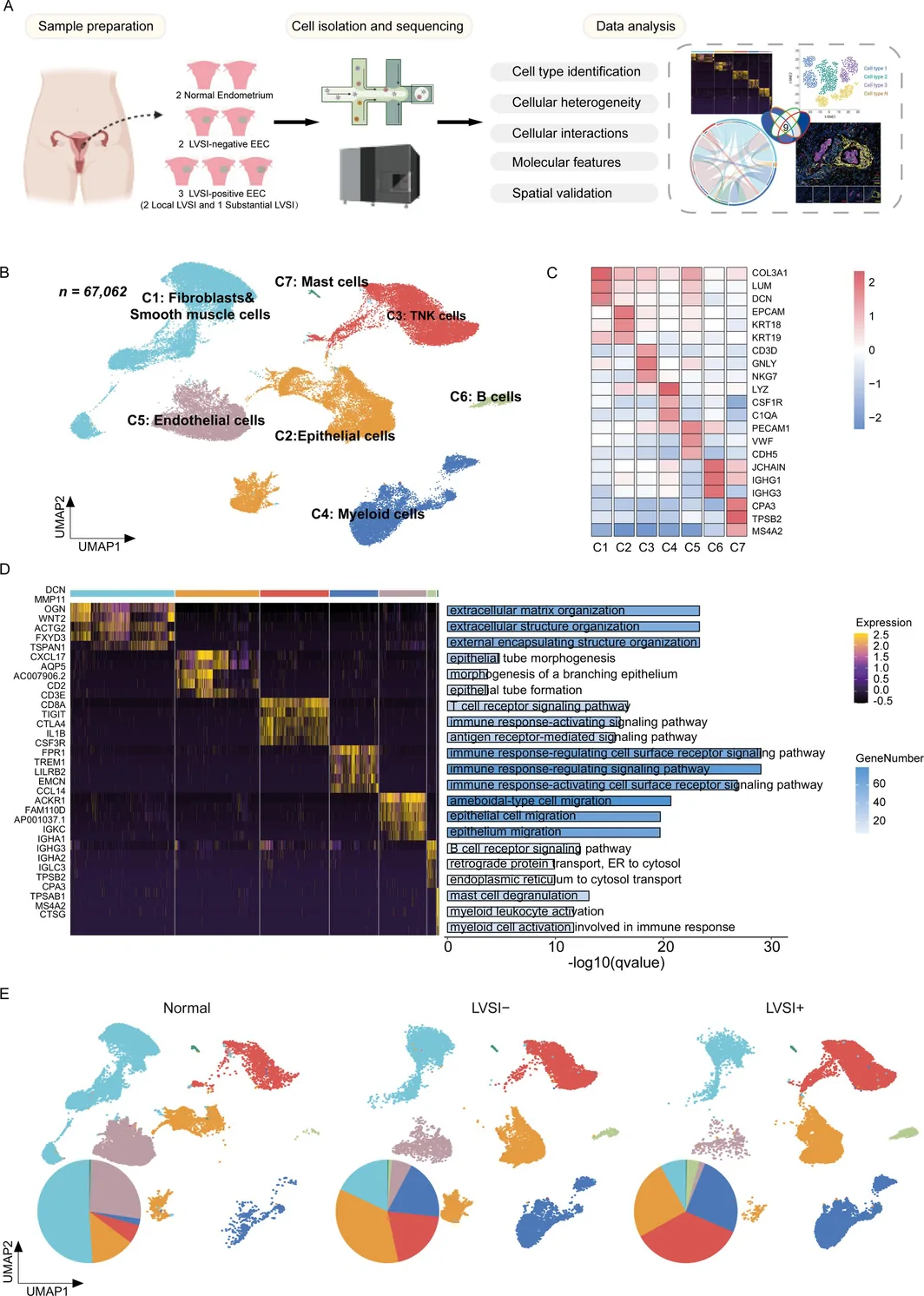
Cellular composition of LVSI+ EEC revealed by scRNA‐seq. (A) Schematic of tissue collection, single‐cell dissociation, scRNA‐seq, and downstream data analysis. (B) UMAP displaying major cell types, with colours representing different cell types. (C) Heatmap of canonical marker genes for each cell type. (D) Heatmap displaying the top 5 upregulated genes (ranked by log2 fold change) for each cell type, with representative genes labelled (left). GO enrichment analysis was performed on these genes, highlighting representative GO terms (right). (E) UMAP split by three tissue origins, with pie charts showing cell‐type proportions. EEC, endometrioid endometrial carcinoma; GO, gene ontology; LVSI, lymphovascular space invasion; LVSI+, LVSI present; scRNA‐seq, single‐cell RNA sequencing; UMAP, uniform manifold approximation and projection.

Using canonical markers, we identified seven major cell types: 15,529 epithelial cells (EPCAM+), 19,249 fibroblasts/muscle cells (COL3A1+), 12,700 T/NK cells (CD3D+), 8590 myeloid cells (LYZ+), 8711 endothelial cells (PECAM1+), 1634 B cells (JCHAIN+), and 289 mast cells (CPA3+) (Figure 1C; Figure S1E). Cell‐type annotations were confirmed by upregulated genes and their biological features (Figure 1D). Compared to LVSI− EEC, LVSI+ EECs showed an enrichment of T/NK cells, myeloid cells, and B cells, with a reduction in fibroblasts and endothelial cells (Figure 1E), consistent with previous studies [22, 23].

### 2.2 Cellular Heterogeneity Across Normal Endometrium, LVSI−, and LVSI+ EEC

Eight epithelial subsets (NC1–2 from normal, TC1–6 from tumour) were identified with a clear separation between normal and tumour epithelia (Figure 2A; Figure S2A). Ciliated TC3 and TC6 cells were dominant in LVSI− EEC, whereas glandular tumour epithelia (TC1, TC2, TC4, TC5) were enriched in LVSI+ EEC (Figure 2B). TC2 and TC5 proportions increased with LVSI progression, and TC5 showed high proliferative activity (Figure 2C; Figure S2B). TC1 and TC4 displayed inflammatory features, including angiogenesis and hypoxia response (Figure S2B).

**FIGURE 2:**
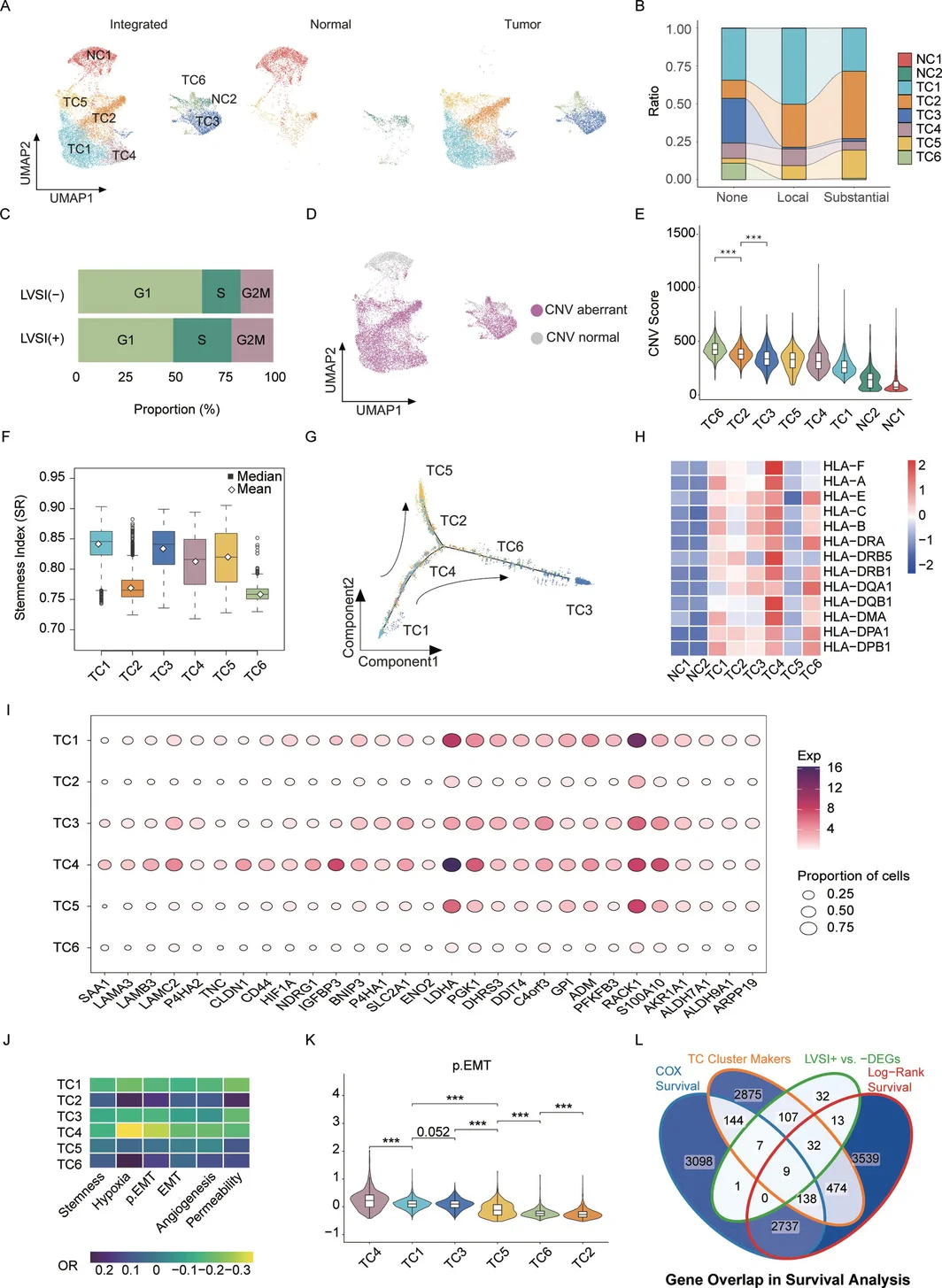
Heterogeneity of epithelial cells in EEC. (A) UMAP visualization of epithelial cells, with colours representing different subsets. (B) Proportions of tumour epithelial cell subsets across different LVSI statuses, with colours indicating distinct subsets. (C) Proportions of cell‐cycle phases in LVSI− and LVSI+ tumour epithelial cells. (D) Identification of malignant epithelial cells using InferCNV. (E) Violin plot showing the distribution of CNV scores across epithelial subsets. (F) Cellular entropy index of epithelial cell subsets. (G) Pseudotime trajectory of tumour epithelial cell differentiation. (H) Expression of MHC class I and class II molecules across epithelial subsets. (I) Bubble plot displaying tumour‐intrinsic programs across tumour epithelial subsets. (J) Heatmap showing tumour behaviour‐related states across tumour epithelial subsets. (K) Violin plot showing the distribution of p‐EMT scores across tumour epithelial subsets. (L) Venn diagram illustrating the overlap of representative prognostic genes derived from epithelial cells. EEC, endometrioid endometrial carcinoma; UMAP, Uniform Manifold Approximation and Projection; LVSI, lymphovascular space invasion; LVSI−, LVSI absent; LVSI+, LVSI present; G1, gap 1 phase; S, synthesis phase; G2/M, gap 2/mitosis phase; CNV, copy number variation; MHC, major histocompatibility complex; p‐EMT, partial epithelial–mesenchymal transition; EMT, epithelial–mesenchymal transition; OR, odds ratio; *p < 0.05; **p < 0.01; ***p < 0.001.

Taking normal epithelium as a reference, tumour epithelium exhibited higher cellular copy number variations (CNVs) than normal epithelium (Figure 2D). The ciliated TC6 subset had the highest CNV burden and the lowest cellular entropy index (Figure 2E,F). Trajectory analysis showed tumour epithelial cells differentiating from TC1/TC4 toward TC3/6 and TC2/5 subsets (Figure 2G).

TC4 expressed higher levels of major histocompatibility complex (MHC) class I and II molecules (Figure 2H). Compared to TC1, TC4 showed upregulation of genes related to partial epithelial–mesenchymal transition (p‐EMT), hypoxia, glycolysis, cytokine signalling, and stemness, and downregulation of progesterone receptor–related genes [24] (Figure 2I, Figure S2C). TC4 demonstrated the strongest anti‐inflammatory, immune‐evasive, and p‐EMT characteristics (Figure 2J,K, Figure S2D). Correlation analysis showed tight associations among hypoxia, inflammation, NF‐κB signalling, angiogenesis, glycolysis, and epithelial–mesenchymal transition (EMT) (Figure S2E).

LVSI− epithelia were enriched in complement and tumour necrosis factor alpha–NF‐κB (TNFA–NFKB) pathways, while LVSI+ epithelia were enriched in MYC/E2F targets and fatty acid metabolic pathways (Figure S2F). By integrating prognostic genes identified from the Cancer Genome Atlas Uterine Corpus Endometrial Carcinoma (TCGA‐UCEC) cohort using univariate Cox regression and log‐rank tests with LVSI‐related differentially expressed genes (DEGs) and epithelial subset‐specific marker genes, we performed a four‐way Venn analysis to identify nine LVSI‐associated prognostic genes, including *HMGA1*, *MGST1*, *NHP2*, *PRDX6*, *RBIS*, *TCEA1*, *TPD52*, *TUFT1*, and *YBX1* (Figure 2L). These genes are functionally linked to chromatin remodelling, redox adaptation, transcriptional regulation, ribosome biogenesis, and proliferative/metastatic programs. Notably, YBX1 is a known oncogenic transcription factor involved in epithelial plasticity and PD‐L1‐associated immune evasion in multiple cancers, supporting its relevance to the aggressive epithelial phenotype in LVSI+ EEC. A summary of the established functions of these nine genes in cancer is provided (Table S1).

### 2.3 T Cell Subsets Shaping the Immunosuppressive TMEM in LVSI+ EEC

T/NK cells were re‐clustered into 11 T‐cell subsets and one NK‐cell subset (Figure 3A,B; Figure S3A,B). Three Cycling_T subsets showed active proliferation (Figure 3C). CD8+ Tem and NK cells were enriched in normal tissues, whereas Cycling_T and ZNF683 + SOX4 + CD8+ T subsets were enriched in LVSI+ EECs (Figure 3D). The ZNF683 + SOX4 + CD8+ T subset was particularly enriched in LVSI‐substantial EEC and the TNFRSF9+ Treg subset was abundant in all EECs (Figure S3C). Cycling_CD8+ T and Cycling_CD4+ T cell frequencies increased with disease progression (Figure 3E).

**FIGURE 3:**
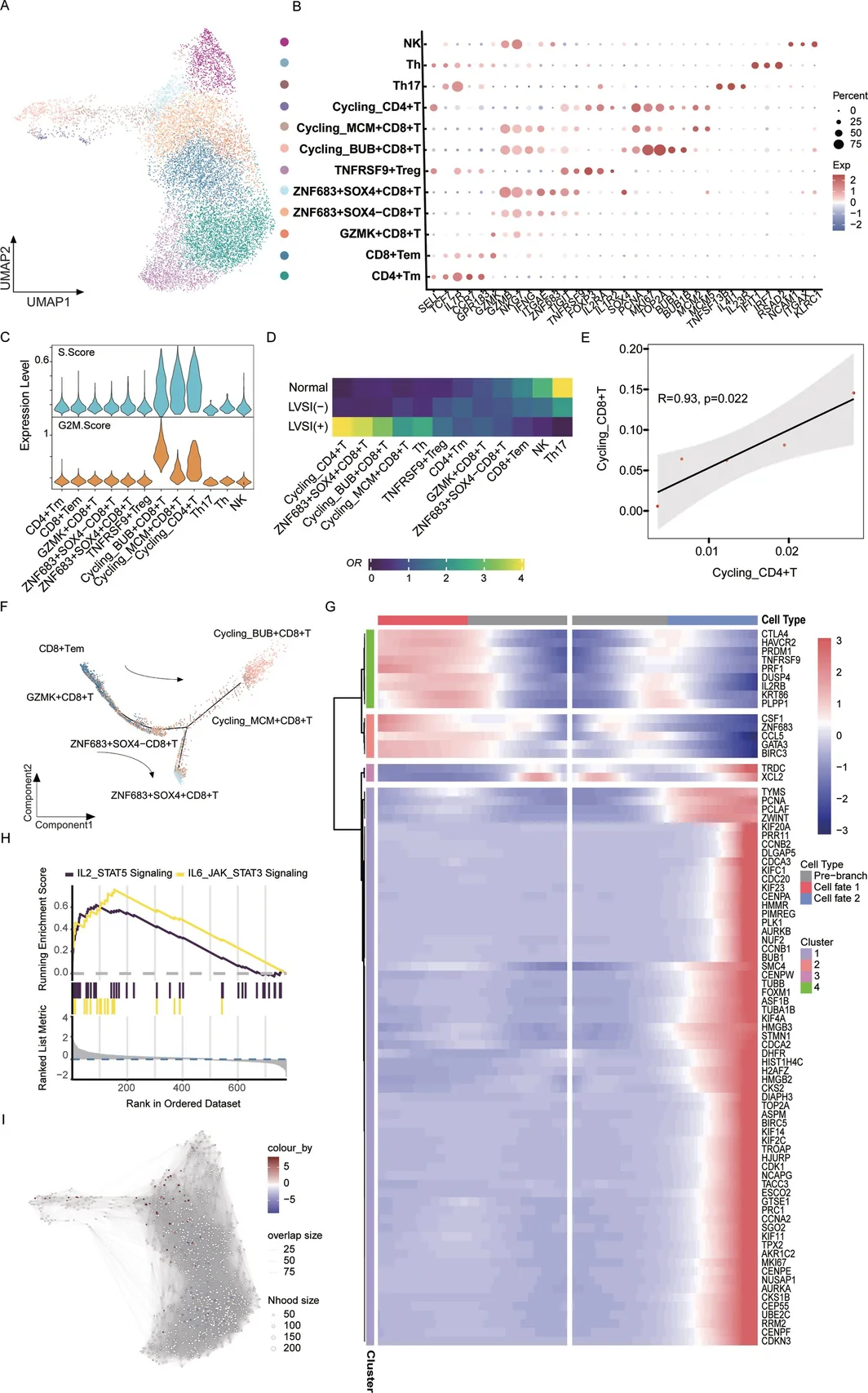
Characterization and functional differentiation of T/NK cell subsets in LVSI+ and LVSI− EEC. (A) UMAP visualization of T/NK cell subsets, with colours representing distinct subsets. (B) Dot plot showing marker gene expression across T/NK subsets, with colour indicating normalized expression level and dot size representing the proportion of expressing cells. (C) Violin plots showing S and G2M phase scores across T/NK subsets. (D) Proportions of T/NK cell subsets in Normal, LVSI−, and LVSI+ samples, with colours indicating odds ratios. (E) Correlation between Cycling_CD8+ T and Cycling_CD4+ T subsets. (F) Pseudotime trajectory of CD8+ T‐cell differentiation. (G) Heatmap showing key genes driving branch decisions along the CD8+ T‐cell trajectory. (H) Pathways upregulated in ZNF683 + SOX4 + CD8 + T cells and TNFRSF9+ Tregs compared with other subsets. (I) Neighbourhood network of T cells between LVSI+ and LVSI− EEC, with node colour ranging from red (LVSI+) to blue (LVSI−). T/NK, T and NK cells; LVSI, lymphovascular space invasion; LVSI+, LVSI present; LVSI−, LVSI absent; EEC, endometrioid endometrial carcinoma; UMAP, Uniform Manifold Approximation and Projection.

CD8+ T cells differentiated from CD8+ Tem into two terminal states: ZNF683 + SOX4 + CD8+ T and Cycling_CD8+ T cells (Figure 3F). Cycling_T cells showed enrichment of proliferation pathways with suppression of inflammatory signalling, while ZNF683 + SOX4 + CD8+ T cells showed enrichment of IL2–STAT5 signalling and inflammatory pathways (Supplementary Figure S3D–F). Branch‐specific gene clustering supported these distinct trajectories (Figure 3G). CD4+ T cells had a similar pattern (Figure S3G,H). Exhausted T cell populations (ZNF683 + SOX4 + CD8+ T and TNFRSF9+ Treg) showed enrichment of STAT5 and STAT3 signalling (Figure 3H). Consistently, T cell neighbourhood network analysis revealed expansion of Cycling_T and ZNF683 + SOX4 + CD8+ T subsets in LVSI+ EECs (Figure 3I).

### 2.4 Myeloid‐Derived Cell Components in LVSI+ EEC

Eleven macrophage and monocyte subsets were identified (Figure 4A,B). APOE‐ and SPP1‐associated subsets, particularly SPP1 + MMP9 + Mac and APOE + CXCL9 + Mac, were enriched in LVSI+ EEC (Figure 4C). APOE‐high macrophages showed stronger antigen‐presentation signatures than SPP1‐high macrophages (Figure 4D). Pseudotime analysis showed differentiation toward APOE + SELENOP + Mac and Proliferative_Mac states (Figure 4E).

**FIGURE 4:**
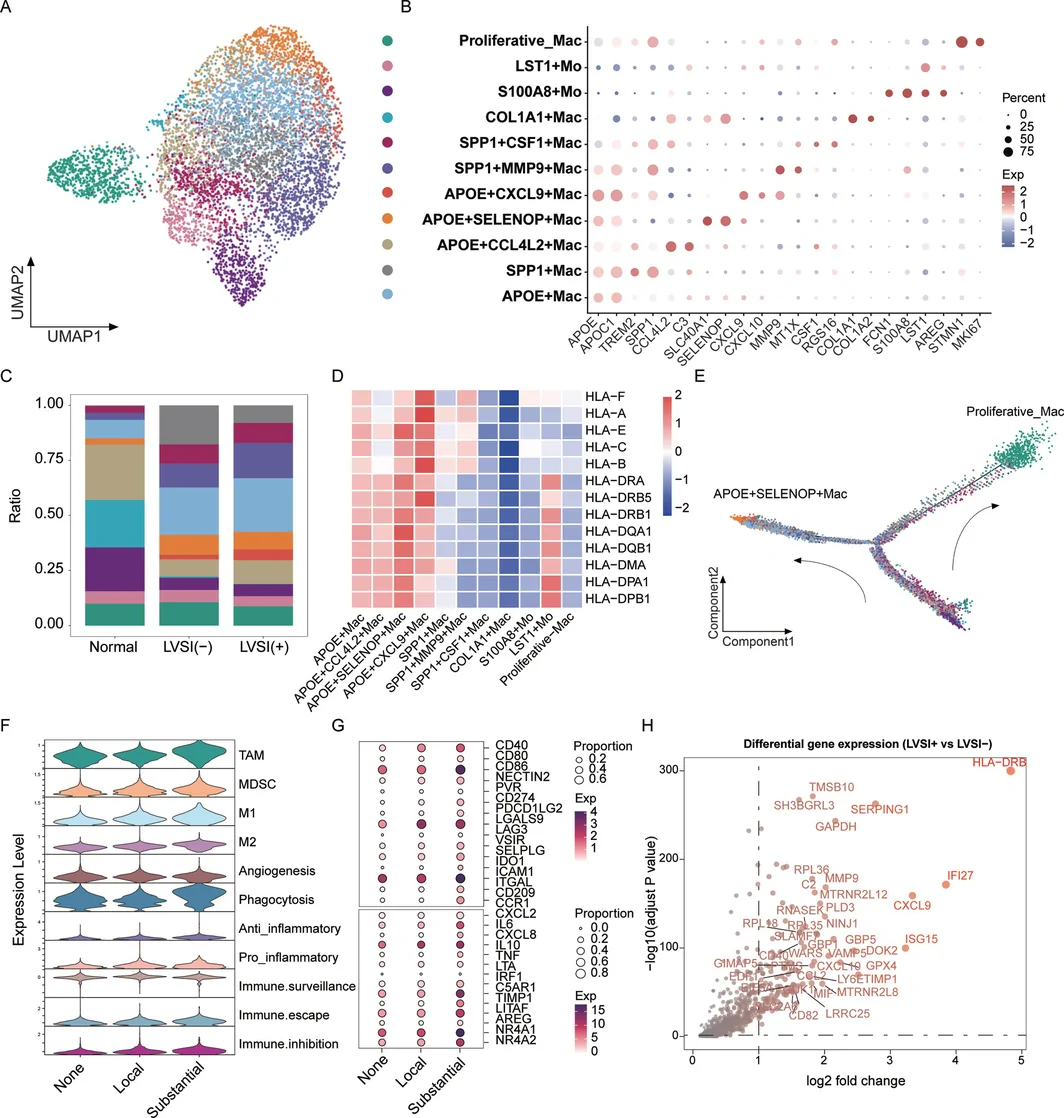
Characterization of monocyte–macrophage heterogeneity in LVSI+ and LVSI− EEC. (A) UMAP visualization of monocyte–macrophage subsets, with colours representing distinct subsets. (B) Dot plot showing marker gene expression across subsets, with colour indicating normalized expression level and dot size representing the proportion of expressing cells. (C) Bar plot showing the proportions of monocyte–macrophage subsets in Normal, LVSI−, and LVSI+ samples. (D) Expression of MHC class I and class II molecules across monocyte–macrophage subsets. (E) Pseudotime trajectory of monocyte–macrophage differentiation. (F) Violin plots showing signature scores under different LVSI states. (G) Bubble plot displaying the expression of signature genes under different LVSI states. (H) Differentially expressed genes between monocyte–macrophage cells from LVSI+ and LVSI− EEC. LVSI, lymphovascular space invasion; LVSI+, LVSI present; LVSI−, LVSI absent; EEC, endometrioid endometrial cancer; UMAP, Uniform Manifold Approximation and Projection; MHC, major histocompatibility complex; TAM, tumour‐associated macrophage; MDSC, myeloid‐derived suppressor cell.

Scores for M1/M2 polarization, tumour‐associated macrophages (TAMs), myeloid‐derived suppressor cells (MDSCs), pro‐angiogenesis, and other functions increased from LVSI− to LVSI+ stages (Figure 4F). Inflammatory chemokines, complement components, immune molecules, and exhaustion molecules showed stepwise upregulation during progression (Figure 4G). All macrophage subsets showed immunosuppressive features, with APOE‐high macrophages exhibiting both high phagocytic activity and TAM scores, whereas SPP1 + MMP9+ macrophages were associated with invasion‐related programs (Figure S4A,B). CXCL9, CXCL10, MMP9, TIMP1, and CCL2 were upregulated in LVSI+ EEC (Figure 4H).

We identified three enriched non‐classical dendritic cell (DC) subsets in LVSI+ EEC: cDC1_LAMP3_DC, cDC2_LAMP3_DC, and pDC (Figure S4C–E). The proportions of cDC2_LAMP3_DC and pDC were strongly positively correlated (Figure S4F). These non‐classical subsets showed low expression of immature DC markers and high expression of mature DC markers [25] (Figure S4G). cDC1_LAMP3_DC displayed high MHC class I and II expression, with MHC class I being further upregulated during the cDC2 to cDC2_LAMP3_DC transition [26] (Figure S4H). Both cDC1_LAMP3_DC and cDC2_LAMP3_DC exhibited strong activation and migratory signatures, whereas Cycling_DC and pDC showed high S and G2/M scores but weak antigen‐presenting capacity (Figure S4H,I).

During LVSI progression, immunosuppressive gene expression in DCs gradually increased (Figure S4J). LAMP3_DC subsets were enriched in interferon and inflammatory signalling pathways and exhibited distinct functions (Figure S4K,L). The differentiation of LAMP3_DC subsets is driven by both shared and subset‐specific gene expression [26] (Figure S4M). In LVSI+ EEC, LAMP3_DC exhibited a state of inflammatory immune activation, engaging TNFA_NFKB, IL6_JAK_STAT3, KRAS, type I interferon (IFN‐α), type II interferon (IFN‐γ) signalling (Figure S4N).

### 2.5 Dissection of Stromal Components and Regulators in LVSI+ EEC

Mesenchymal cells were re‐clustered into 8 fibroblast and 1 myocyte subset (Figure 5A,B). The iCAF, WNT5A_mCAF, LRRC75A_mCAF, and dCAF subsets were enriched in LVSI+ EEC (Figure 5C). These cancer‐associated fibroblast subpopulations displayed distinct functional programs (Figure 5D,E). WNT5A_mCAF and iCAF showed high expression of genes related to hypoxia, pEMT, and inflammation (Figure 5F). WNT5A_mCAF and dCAF represented two distinct differentiation trajectories (Figure 5G). WNT5A_mCAF was enriched in coagulation, hypoxia, angiogenesis, and EMT pathways (Figure 5H), with distinct transcription factor activation patterns across subsets (Figure 5I). Upregulated genes in LVSI+ versus LVSI− EEC were enriched in pathways related to IFN‐γ signalling, collagen biosynthesis, lymphocyte apoptosis, macrophage migration, and epithelial sprouting (Figure 5J). During LVSI progression, the microenvironment shifts toward an anti‐inflammatory, pro‐angiogenic, and immunosuppressive state (Figure 5K).

**FIGURE 5:**
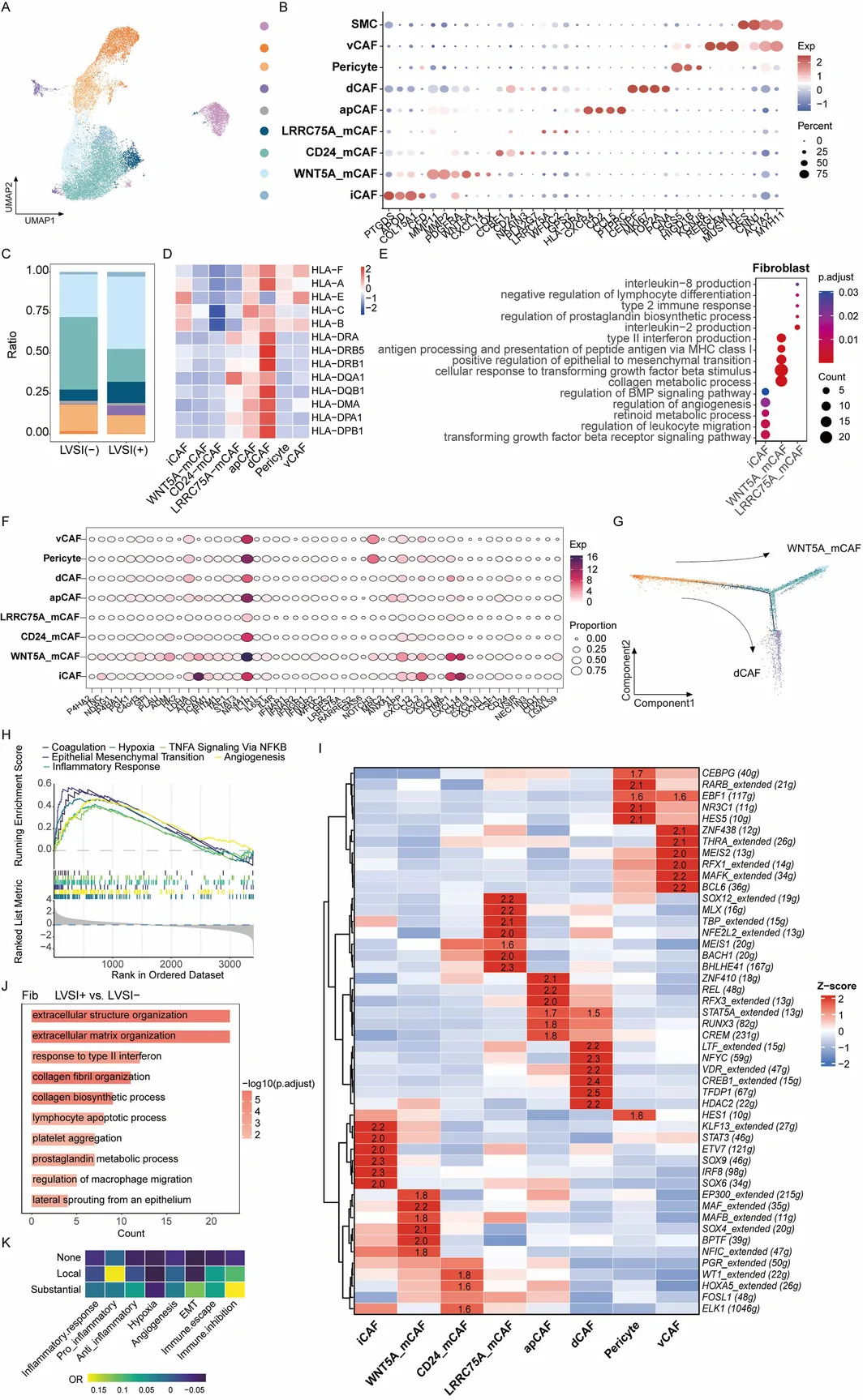
Characterization of fibroblast heterogeneity in LVSI+ and LVSI− EEC. (A) UMAP visualization of fibroblast subsets, with colours representing distinct subsets. (B) Dot plot showing marker gene expression across fibroblast subsets, with colour indicating normalized expression level and dot size representing the proportion of expressing cells. (C) Bar plot showing the proportions of fibroblast subsets in LVSI− and LVSI+ EEC. (D) Expression of MHC class I and class II molecules across fibroblast subsets. (E) Bubble plot displaying pathways enriched in iCAF, WNT5A_mCAF, and LRRC75A_mCAF subsets. (F) Dot plot showing the expression of signature genes across fibroblast subsets. (G) Pseudotime trajectory of fibroblast differentiation. (H) Pathways significantly upregulated in the WNT5A_mCAF subset compared with other fibroblast subsets. (I) Heatmap showing key transcription factors across fibroblast subsets. (J) Bar plot showing pathways enriched in LVSI+‐upregulated genes relative to LVSI− EEC. (K) Heatmap showing signature scores across different LVSI states. LVSI, lymphovascular space invasion; LVSI+, LVSI present; LVSI−, LVSI absent; EEC, endometrioid endometrial carcinoma; UMAP, Uniform Manifold Approximation and Projection; MHC, major histocompatibility complex; EMT, epithelial–mesenchymal transition.

Endothelial cells (ECs) were classified into 12 subsets, including 8 vascular, 3 endothelial‐to‐mesenchymal transition (EndoMT), and 1 lymphatic subset (Figure 6A,B). Tumour‐enriched ECs clustered predominantly within three tip endothelial subsets (Tip_ECs): Tip_ArtECs, Tip_CapECs, and Tip_VenECs (Figure 6C). Both Cycling_ECs and Tip_ECs were at terminal stages of vascular endothelial differentiation (Figure 6D). Tip_ECs mainly expressed MHC class I molecules, whereas mature ECs expressed both MHC class I and II (Figure 6E). Tip_ECs were significantly enriched in angiogenesis, EMT, and TNFA–NFKB signalling pathways (Figure 6F). Functionally, all three Tip_EC subsets were pro‐inflammatory, with Tip_ArtECs driving angiogenesis and Tip_VenECs promoting EMT (Figure 6G).

**FIGURE 6:**
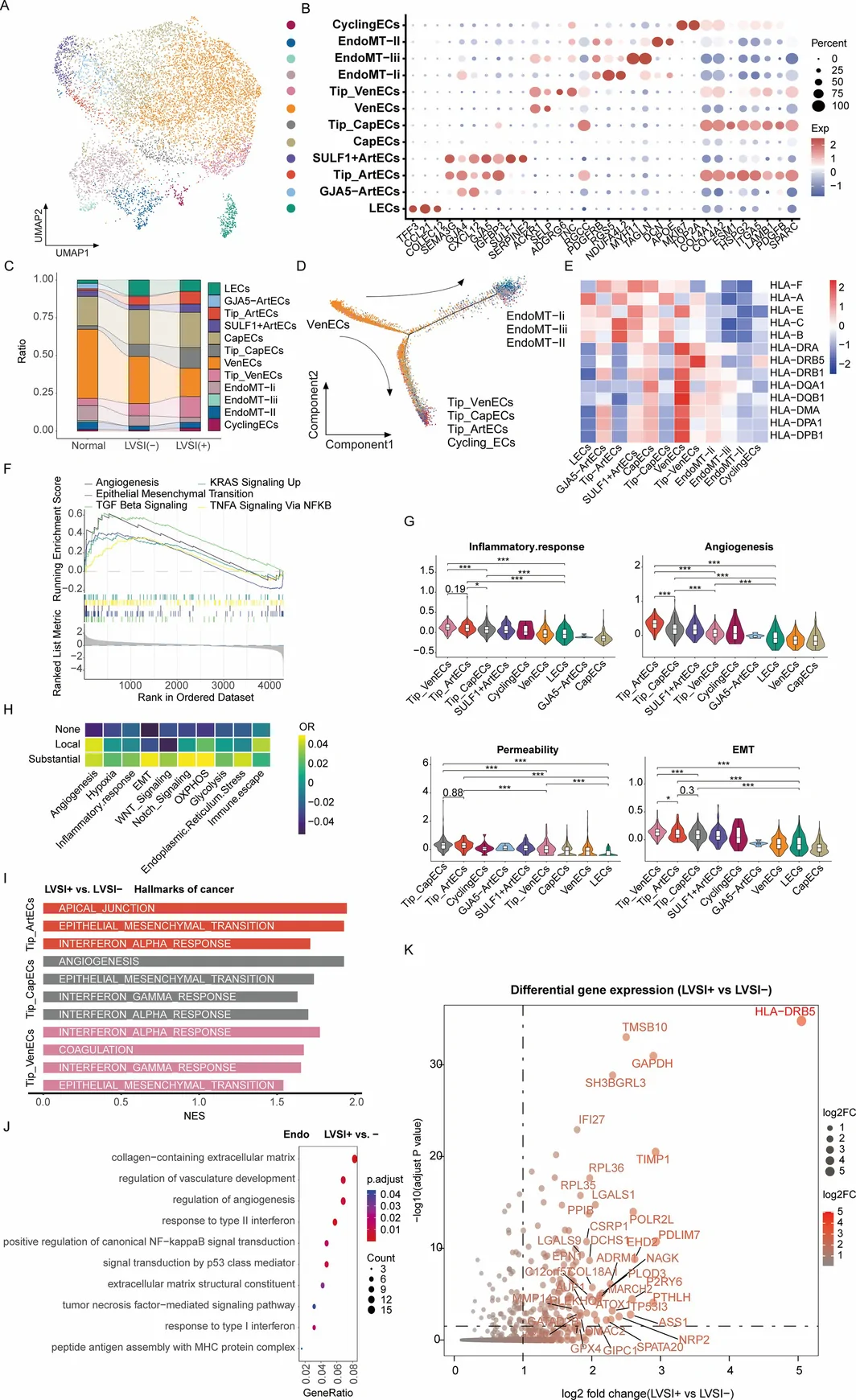
Endothelial cell heterogeneity and functional features in LVSI+ and LVSI− EEC. (A) UMAP visualization of endothelial subsets. (B) Dot plot showing marker gene expression across subsets, with colour indicating normalized expression level and dot size representing the proportion of expressing cells. (C) Proportions of endothelial subsets in Normal, LVSI−, and LVSI+ samples. (D) Pseudotime trajectory of endothelial cell differentiation. (E) Expression of MHC class I and class II molecules across endothelial subsets. (F) Pathways upregulated in Tip_ECs subsets relative to other endothelial subsets. (G) Violin plots showing inflammation, angiogenesis, permeability, and EMT scores across endothelial subsets. (H) Heatmap showing signature scores under different LVSI states. (I) Differentially enriched pathways in Tip_ArtECs, Tip_CapECs, and Tip_VenECs between LVSI+ and LVSI− EEC. (J) Bubble plot showing pathways enriched in genes upregulated in LVSI+ versus LVSI− EEC. (K) Differentially expressed genes between endothelial cells from LVSI+ and LVSI− EEC. LVSI, lymphovascular space invasion; LVSI+, LVSI present; LVSI−, LVSI absent; EEC, endometrioid endometrial carcinoma; UMAP, Uniform Manifold Approximation and Projection; MHC, major histocompatibility complex; EMT, epithelial–mesenchymal transition; OXPHOS, oxidative phosphorylation; *p < 0.05; **p < 0.01; ***p < 0.001.

Compared to LVSI‐local EEC, pathways for hypoxia, inflammatory response, EMT, NOTCH signalling, oxidative phosphorylation, and endoplasmic reticulum stress [27] were most activated in LVSI‐substantial EEC (Figure 6H). In both LVSI+ and LVSI− EEC, all Tip_EC subsets were enriched for EMT and IFN‐α signalling (Figure 6I). Genes upregulated in LVSI+ versus LVSI− EEC were enriched in pathways involving extracellular matrix remodelling, angiogenesis, interferon, and inflammatory response (Figure 6J). In LVSI+ EEC, ECs showed increased permeability (*PLVAP*), enhanced immunosuppressive signalling (*LGALS9*), basement membrane remodelling (collagens), and altered migratory behaviour (MMPs) (Figure 6K).

### 2.6 Identification of a Multicellular Community Driving the TMEM in LVSI+ EEC

Co‐enrichment analysis showed that TC4 clustered with cDC1_LAMP3_DC and several macrophage subsets (Figure 7A). In the cell–cell communication network, LVSI− EEC epithelia were main signalling hubs, while in LVSI+ EEC, fibroblasts, endothelial cells, and myeloid cells became the dominant interactors (Figure 7B). Ligands upregulated in LVSI+ EEC were associated with tumour promotion, angiogenesis, and metastatic pathways and were mainly derived from stromal components, whereas ligands in LVSI− EEC were enriched in cytokine and inflammatory pathways (Figure 7C).

**FIGURE 7:**
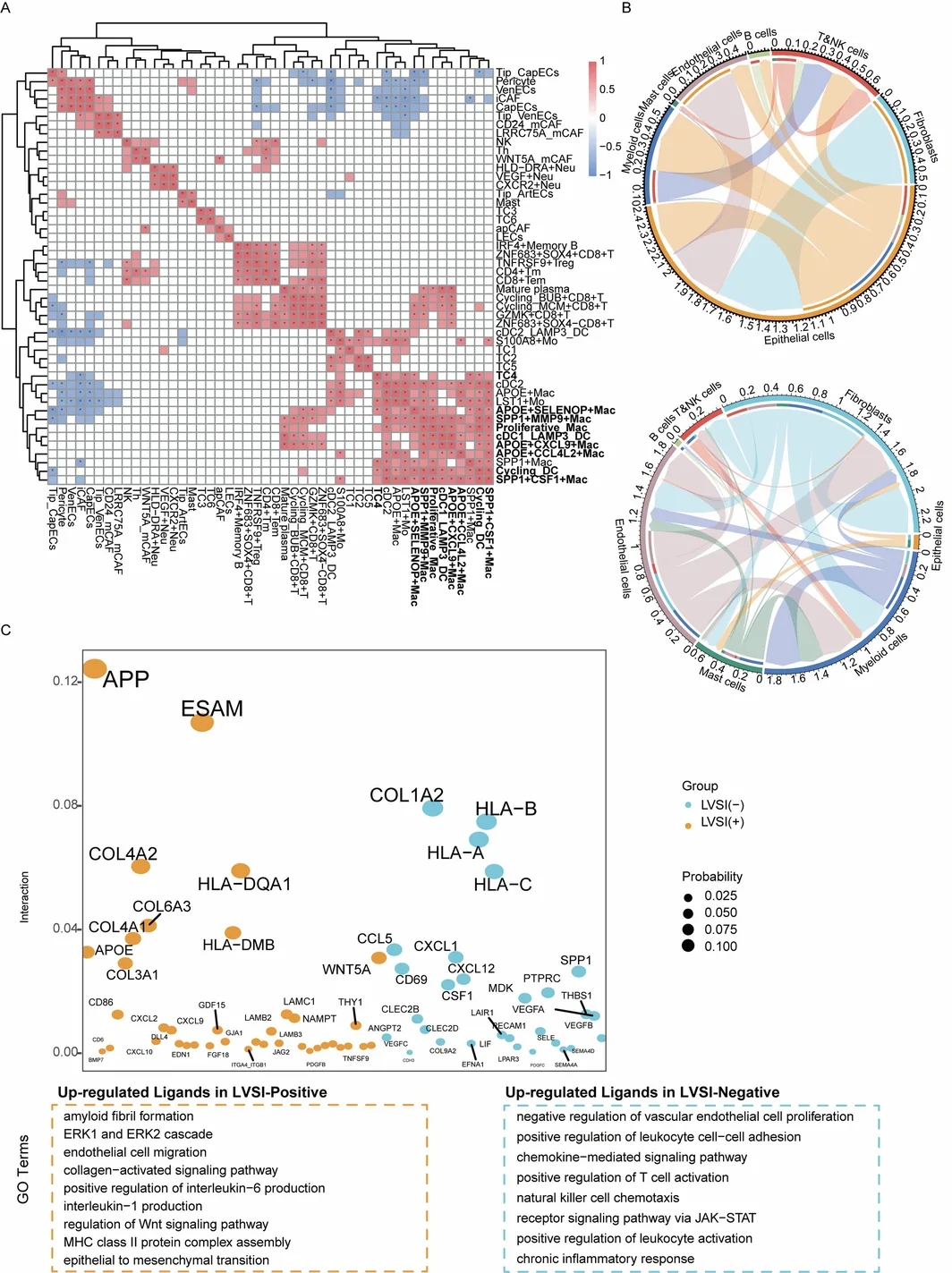
Distinct cell–cell interaction patterns in the LVSI+ EEC tumour microenvironment. (A) Heatmap showing correlations among tumour, immune, and stromal cell subset proportions. *p* < 0.05 was used as the cutoff. (B) Chord diagrams showing the interactions among major cell types in LVSI− (top) and LVSI+ EEC (bottom). (C) Scatter plot showing the specific ligands in LVSI+ and LVSI− EEC (top) and their enriched pathways (bottom), with colour indicating tissue origin and dot size representing interaction probability. LVSI, lymphovascular space invasion; LVSI+, LVSI present; LVSI−, LVSI absent; EEC, endometrioid endometrial carcinoma; GO term, gene ontology term.

LVSI+ EEC–specific ligands showed DLL4/JAG2 activation in endothelial cells [28], high ligand expression (CXCL9, APOE) in macrophages, and WNT5A/BMP7 activation in fibroblasts (Figure S5A). In LVSI+ EEC, WNT5A_mCAF and SPP1 + MMP9 + Mac were key signal senders and receivers, in contrast to neutrophils and tumour cells in LVSI− EEC (Figure S5B). SPP1 + MMP9 + Mac primarily communicated with LECs and Tip_CapECs, while WNT5A_mCAF interacted with Tip_ArtECs and Tip_VenECs. Both populations showed close communication with exhausted T cell subsets (Figure S5C).

In LVSI− EEC, epithelial cells signal through the PLAU–PLAUR axis to interact with various immune and stromal cells. Furthermore, epithelial cells release chemokines that activate Tip_ECs via the ACKR1 receptor and recruit neutrophils via CXCR receptors [29, 30] (Figure S6A). In LVSI+ EEC, SPP1 + MMP9 + Mac targeted LECs/Tip_ECs via the SPP1–integrin axis, which is known to promote endothelial cell adhesion and migration and likely facilitates the docking and activation of lymphatic endothelial cells, creating a permissive channel for tumour cell intravasation. Meanwhile, WNT5A_mCAF promoted angiogenesis through collagen–integrin and WNT5A–MCAM signalling, enhancing endothelial motility and extracellular matrix remodelling, thereby contributing to LVSI progression (Figure S6B). These populations also suppressed T/NK cell function via LGALS9 signalling axes (Figure S6C). LECs and Tip_ECs also induced T cell exhaustion (Figure S6D).

Multiplex immunofluorescence revealed a pro‐metastatic microenvironment at the invasive front characterized by hypoxia, proliferation, and immunosuppression. Tumour epithelial cells in this region displayed high HIF1A expression, consistent with the TC4 subset (Figure S6). With LVSI progression, CD44 expression shifted from epithelial to stromal immune cells, and PD‐L1+ macrophages redistributed from the tumour stroma and glandular lumen to the peritumoral stroma and surrounding invasive tumour clusters (Figures S7 and S8).

In LVSI‐substantial EEC, CD4+ and CD8+ T cells accumulated around invasive tumour epithelium with increased PD‐1 expression (Figure S9). In addition, CD4 and CD8 T‐cell subsets showed co‐expression of Ki67 and PD‐1, consistent with the exhausted Cycling_T subsets (Figure S9). Spatial colocalization of PD‐L1+ tumour cells, PD‐L1+ macrophages, and PD‐1+ T cells was observed within tumour emboli (Figure 8). A model of this crosstalk was generated (Figure S10).

**FIGURE 8:**
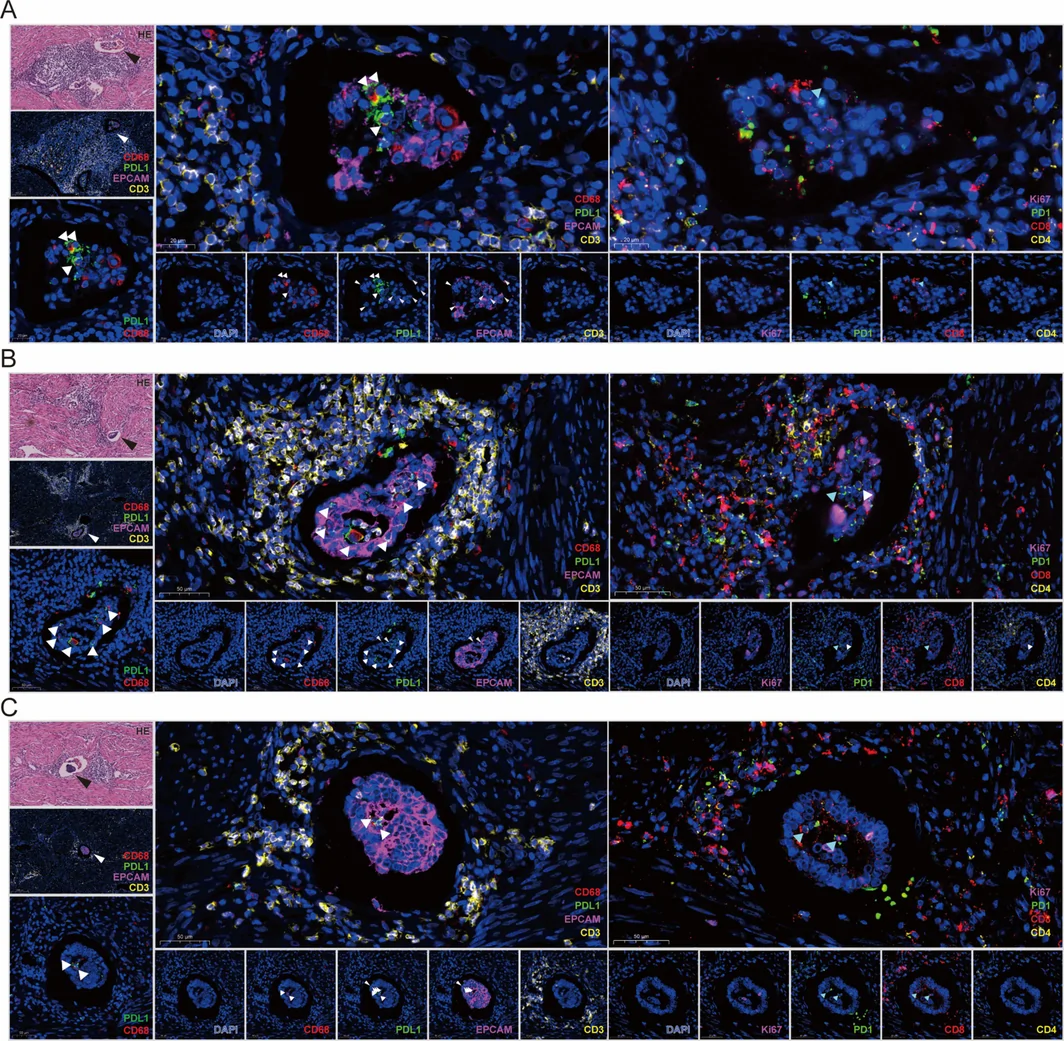
Spatial colocalization of immune checkpoint–associated cells within tumour emboli in LVSI‐substantial EEC. Representative HE and mIF images showing the spatial distribution and colocalization of PD‐L1+ malignant epithelial cells, PD‐L1+ macrophages, and PD‐1+ T cells within tumour emboli in LVSI‐substantial EEC. Panels A–C represent distinct LVSI structures at different spatial locations. Black arrows in the upper‐left first row (HE) and white arrows in the second row (mIF) indicate the regions magnified on the right. Wide and narrow white arrows denote PD‐L1 co‐expression with CD68 and EPCAM, respectively. Cyan and dark yellow short arrows indicate PD‐1 co‐expression with CD8 and CD4, respectively. Nuclei are counterstained with DAPI. Scale bars are indicated. LVSI, lymphovascular space invasion; EEC, endometrioid endometrial carcinoma; HE, haematoxylin–eosin; mIF, multiplex immunofluorescence; PD‐1, programmed cell death protein 1; PD‐L1, programmed death‐ligand 1.

## 3 Discussion

Lymphovascular space invasion is a critical prognostic factor in endometrioid endometrial cancer, increasing metastasis risk and guiding clinical decisions. While its pathological role is clearly defined, the underlying multicellular and molecular mechanisms remain unclear. Our study combined single‐cell transcriptomics with spatial and clinical validation to analyse LVSI‐present tumours, revealing a spatially coordinated pro‐invasive network among malignant epithelial, immune, and stromal cells. We found epithelial cells transition dynamically from ciliated differentiation to proliferation, while the tumour microenvironment undergoes functional reprogramming, including extracellular matrix remodelling, angiogenesis, and immunosuppression. This ecosystem actively drives lymphovascular space invasion, suggesting new biomarkers and microenvironment‐targeted therapies for high‐risk endometrioid endometrial cancer.

In terms of epithelial heterogeneity, this study challenges the traditional notion that EEC originates exclusively from non‐ciliated glandular epithelium [23]. TC6 ciliated cells exhibit high CNV levels and low cellular entropy, and trajectory analysis identifies TC1 and TC4 as initial malignant states that give rise to both ciliated (TC3 and TC6) and proliferative (TC2 and TC5) lineages. Among these, TC4 represents a key invasive subset, characterized by high expression of *HIF1A*, *CD44*, *PLAU*, and *SAA1*, and enriched for p‐EMT, EMT, hypoxia, angiogenesis, inflammation, NF‐κB signalling, glycolysis, and immunosuppression, consistent with its role in the progression from atypical hyperplasia to carcinoma [23]. A strong correlation between p‐EMT scores and malignancy grade is also observed across different pathological subtypes of endometrial cancer [31]. In addition, the prometastatic state of TC4 is accompanied by downregulation of progesterone receptor–related genes, indicating progesterone resistance. Collectively, during the transition from LVSI− to LVSI+ stages, malignant epithelial cells shift from differentiated states toward a metastatic proliferative phenotype driven by persistent inflammation, hypoxia, and fatty‐acid metabolic reprogramming in EEC.

A central conclusion of this study is that LVSI is not driven by a single cellular source but emerges from coordinated interactions among multiple tumour microenvironment components. In LVSI+ EEC, cell–cell communication shifts from epithelium‐directed recruitment of inflammatory neutrophils and macrophages toward dominant immune–stromal crosstalk, promoting a transition from an inflammatory to a synergistic pro‐metastatic microenvironment [32]. At the tumour boundary, extensive extracellular matrix reconstruction and immunomodulatory remodelling result in spatial invasion and molecular immunosuppression. Spatially, WNT5A_ mCAF remodel the extracellular matrix and create a permissive stromal niche that supports EMT and enables angiogenesis and lymphangiogenesis through collagen deposition and WNT5A ligand signalling [33]. Further, SPP1+ MMP9+ Mac directly engage LECs and Tip_ECs via SPP1–integrin signalling to initiate endothelial activation and angiogenic sprouting [34, 35, 36, 37], while simultaneously contributing to fibrotic remodelling through the SPP1–CD44 axis [38]. These two cell populations co‐localize with malignant epithelium at the invasive front, forming a pan‐cancer immune‐excluding barrier [33, 39] that promotes tumour progression and impairs immunotherapy responses [40, 41, 42, 43].

At the molecular level, this study demonstrates that SPP1+ MMP9+ Mac, WNT5A_mCAF, LECs, and Tip_ECs impair T cell function through LGALS9 signalling. Similar immunosuppressive interactions have been reported across different cancers [15, 33, 42, 44]. Distinct from LVSI− and LVSI‐local EEC, multiplex immunofluorescence in LVSI‐substantial EEC reveals significant T cell enrichment at the invasive front, where proliferative T cells co‐express Ki67 and PD‐1, indicating a pre‐exhaustion phenotype similar to that observed in clear cell renal cell carcinoma [45] and hepatocellular carcinoma with microvascular invasion [16]. Furthermore, PD‐L1+ malignant epithelial cells, PD‐L1 + Mac cells, and PD‐1+ T cells co‐localize within vascular tumour thrombi, forming spatially organized immunosuppressive units. Consistent with real‐time imaging studies, macrophages can be educated by tumour cells to become key facilitators of vascular metastasis, with TIE2hi macrophages transiently increasing vascular permeability to promote tumour cell intravasation [46]. Collectively, a spatially coordinated multicellular immunosuppressive network, combined with immunosuppressive signals from the sprouting vasculature [47], drives malignant epithelial cell vascular invasion.

This study provides direct translational insights for precision management of EEC. At the diagnostic level, the LVSI‐associated prognostic genes derived from malignant epithelial and LVSI‐associated subsets may serve as biomarkers to predict LVSI status in preoperative biopsies. These markers are particularly valuable for patients with early‐stage disease where imaging diagnosis is limited yet metastatic potential is high, or for those with myometrial invasion who desire conservative treatment, thus providing molecular guidance to avoid under‐ or overtreatment. Therapeutically, among the candidate axes, the LGALS9‐centred inhibitory network appears particularly compelling because it is deployed by multiple cellular compartments in LVSI+ EEC, including macrophages, fibroblasts, and endothelial cells, and converges on dysfunctional T‐cell states. This suggests that blockade of LGALS9‐related signalling may be especially valuable when combined with PD‐1/PD‐L1 inhibition to overcome layered immune suppression. In parallel, the SPP1–integrin axis and WNT5A‐associated stromal signalling represent attractive strategies for disrupting extracellular matrix remodelling, endothelial activation, and the formation of permissive invasive niches [48, 49]. Additional targeting of neutrophil responses [50, 51], and endothelial programs [47] may further limit inflammatory infiltration and vascular invasion. Collectively, these findings support the prioritized multi‐component, microenvironment‐directed strategies to improve outcomes in LVSI+ EEC.

This study provides the first high‐resolution single‐cell atlas of LVSI‐present endometrioid endometrial carcinoma, moving beyond previous mechanistic descriptions [20] to delineate the complex cellular ecosystem and multicellular networks that drive lymphovascular space invasion. These findings transform LVSI from a purely pathological phenotype into a targetable biological process driven by specific cell interactions, thereby supporting the development of combination therapeutic strategies and novel predictive tools. Nevertheless, several limitations of our study should be acknowledged. Our foundational findings, derived from a discovery cohort of five EEC patients, require validation in larger, independent cohorts stratified by molecular subtype to assess generalizability. While our mIF validation provides spatial protein context, future studies integrating scRNA‐seq with spatial transcriptomics on the same LVSI+ specimens would allow direct transcriptional mapping of the coordinated niches we propose. Further large‐scale studies will be required to confirm the nine LVSI‐associated prognostic genes at the protein level using multiplex immunofluorescence or immunohistochemistry and to validate findings in younger patient cohorts to support fertility‐sparing management and clinical translation.

In summary, this study systematically characterizes the cellular landscape and molecular features of LVSI‐present endometrioid endometrial carcinoma, thereby providing key insights into improved diagnosis, risk stratification, and treatment strategies.

## 4 Materials and Methods

### 4.1 Ethical Approvement and EC Samples Collection

This study was approved by the Medical Ethics Committee of Tianjin Central Hospital of Gynaecology Obstetrics (Approval No.: 2024KY079). All samples were from treatment‐naïve patients. Five patients with EC (FIGO 2009 stage IA EEC) provided hysterectomy samples, and two normal endometrial tissues were obtained from patients with uterine leiomyomas (Table S2). LVSI status was independently confirmed by two pathologists using D2‐40/EPCAM and CD31/EPCAM staining and was classified as “substantial” when ≥ 5 distinct vascular channels contained tumour emboli or when a confluent pattern was observed, as per established criteria; otherwise, it was classified as “local”. Pathologists collected all scRNA‐seq samples to ensure tissue freshness and consistency. For validation, formalin‐fixed, paraffin‐embedded (FFPE) sections from four LVSI− and four LVSI+ EEC cases (two LVSI‐local and two LVSI‐substantial) were used for staining (Table S3).

### 4.2 Human Endometrial Tissue Dissociation and Preparation for scRNA Sequencing

Fresh tissues were dissociated using the SeekMate Tissue Dissociation Reagent Kit A Pro (SeekGene). Following viability assessment, single‐cell RNA‐seq libraries were prepared with the SeekOne Digital Droplet Single Cell 3′ Kit and sequenced on an Illumina NovaSeq 6000 (PE150 reads).

### 4.3 Preprocessing the scRNA‐Seq Data

Raw sequencing data were processed using Fastp [52] to trim low‐quality bases and primer sequences. Subsequently, SeekOneTools was used for alignment to the human GRCh38 genome to generate the gene expression matrix.

### 4.4 Quality Control, Integration, and Clustering of scRNA‐Seq Data

Using Seurat (v5.2.0, https://satijalab.org/seurat/), cells with fewer than 200 or more than 7500 detected genes, or with mitochondrial gene percentages exceeding 15%, were filtered. Data were normalized and scaled. Datasets were integrated using IntegrateLayers with HarmonyIntegration. Clustering was performed on the harmony reduction using FindClusters (resolution = 0.01) and visualized with RunUMAP. The final dataset contained 67,062 cells and 30,421 genes.

### 4.5 Cell Type Annotation

In addition to identifying 66 distinct cell clusters, multiple layers of detail were incorporated to achieve a comprehensive understanding of cellular heterogeneity. Cell type annotation was guided by canonical marker gene expression from prior studies [26, 53, 54, 55, 56, 57, 58, 59, 60, 61, 62, 63, 64, 65, 66, 67, 68] and distinctive features in UMAP embeddings.

### 4.6 Tissue Distribution Preferences and Differential Abundance

Tissue distribution was assessed using odds ratio–based tests. Differential abundance was evaluated with the miloR framework (https://github.com/MarioniLab/miloR) by constructing a *k*‐nearest neighbour graph, defining neighbourhoods, and applying a generalized linear model.

### 4.7 InferCNV Analysis for Malignant Cell Identification

The InferCNV R package (https://github.com/broadinstitute/infercnv) was used to distinguish malignant from normal epithelial cells (NC1 and NC2 as reference). The analysis was run with a cutoff of 0.1 and denoising enabled. A CNV score for each cell was derived by summing aberrant genomic regions to classify cells as malignant.

### 4.8 Identification of DEGs and Functional Enrichment

DEGs were identified using Seurat's FindMarkers (https://satijalab.org/seurat/reference/findmarkers) function (adjusted *p* value < 0.05, |log_2_FC| > 1). Gene Set Enrichment Analysis (GSEA) was performed with MSigDB HALLMARK gene sets (https://www.gsea‐msigdb.org/gsea/index.jsp) [69], and Gene Ontology (GO) analysis was conducted with the enrichGO function [70].

### 4.9 Single‐Cell Entropy Analysis

Cellular stemness was assessed by quantifying signalling entropy with the LandSCENT R package (https://github.com/ChenWeiyan/LandSCENT). Entropy values were computed using the CompSRana function, and cell potency states were classified using the InferPotency function.

### 4.10 Pseudotime Trajectory

Pseudotime trajectory analysis was performed using Monocle2 (https://github.com/cole‐trapnell‐lab/monocle‐release) [71]. A CellDataSet object was created from normalized counts, and the trajectory was constructed using the DDRTree method. Branch expression analysis was conducted to identify genes associated with lineage bifurcations.

### 4.11 Gene Signature Scoring Analysis

Functional enrichment was quantified using Seurat's AddModuleScore function (https://satijalab.org/seurat/reference/addmodulescore) [72] for predefined gene signatures [26, 73, 74, 75, 76, 77, 78, 79] and by calculating mean z‐score–normalized expression for heatmap and UMAP visualization.

### 4.12 Survival Analysis and Gene Set Intersection

Data were obtained from the TCGA‐UCEC database (UCSC Xena portal). Prognostic genes were identified using univariate Cox regression and log‐rank tests, based on overall survival. Subset‐specific marker genes and LVSI‐related DEGs were identified using predefined statistical criteria and integrated through a four‐way Venn analysis, retaining only overlapping genes as final LVSI‐associated prognostic candidates.

### 4.13 Transcription Factor Regulatory Networks

The SCENIC package (https://github.com/aertslab/SCENIC) [80] was used to reconstruct transcription factor (TF) regulatory networks in fibroblasts. The pipeline involved inferring co‐expression networks (GENIE3), constructing regulons (RcisTarget), and quantifying TF activity (AUCell). The resulting TF activities were visualized through heatmap displays, showing regulon activation patterns across different cell subtypes.

### 4.14 Cell–Cell Communication Analysis

The CellChat R package (https://github.com/jinworks/CellChat) was used to analyse communication networks. Ligand–receptor interactions were identified from the CellChatDB database. Centrality and comparative analyses were performed to identify key communicators and differential interactions between LVSI+ and LVSI− groups.

### 4.15 Histopathology and Multiplex Immunofluorescence

4‐μm‐thick FFPE sections were processed for Haematoxylin–eosin staining and multiplex immunofluorescence (mIF). For mIF, sections underwent antigen retrieval and sequential tyramide signal amplification using three panels: Panel 1: anti‐Ki67 (Huabio, #HA721115, 1:5000), anti‐HIF1A (Zenbio, #340462, 1:300), anti‐EPCAM (Abcam, #ab213500, 1:10000), anti‐CD44 (Abcam, #ab189524, 1:4000); Panel 2: anti‐CD68 (Huabio, #HA601115, 1:1000), anti‐PD‐L1 (Huabio, #HA721176, 1:500), anti‐EPCAM (Abcam, #ab213500, 1:10000), anti‐CD3 (Abcam, #ab237721, 1:2000); Panel 3: anti‐Ki67 (Huabio, #HA721115, 1:5000), anti‐PD‐1 (Abcam, #ab243644, 1:500), anti‐CD8 (Abcam, #ab237709, 1:4000), anti‐CD4 (Huabio, #ET1609‐52, 1:1000). Nuclei were labelled with DAPI, and slides were imaged using a Pannoramic 250 FLASH III scanner.

### 4.16 Statistical Analysis

Continuous variables were summarized as mean ± standard deviation (SD). The Wilcoxon rank‐sum test was used for two‐group comparisons, and one‐way ANOVA for multi‐group comparisons. Analyses were performed using R (v4.3.3) and all tests were two‐sided. Statistical significance was denoted as *p* < 0.05 (*), *p* < 0.01 (**), and *p* < 0.001 (***).

## Author Contributions

W.G. and Y.H. conceived the study. W.G., N.L., J.S., and R.R. collected samples and collected data. Y.P. and W.Z. contributed to study design. W.G., T.L., Y.P., and R.R. performed analysis. W.G. prepared figures and drafted the manuscript. Y.H. supervised the study. All authors reviewed and approved the final manuscript.

## Funding

This study was supported by grants from the Tianjin Education Commission Scientific Research Project (2023YXZD06), the Tianjin Municipal Health and Science Technology Project (TJWJ2025XK010), the Tianjin Science and Technology Program (23JCZDJC00810), and the Tianjin Key Medical Discipline (Specialty) Construction Project (TJYXZDXK‐3‐029C).

## Ethics Statement

The Ethics Committee of Tianjin Central Hospital of Gynaecology Obstetrics (Approval No.: 2024KY079) approved this study. Procedures followed the Declaration of Helsinki. Written informed consent was obtained from all patients.

## Conflicts of Interest

The authors declare no conflicts of interest.

## Supporting information


**Figure S1:** Data quality control and filtering. (A) HE staining of samples derived from seven patients. (B) Violin plots illustrating nFeature_RNA, nCount_RNA, and mitochondrial gene expression (percent mt) before (top) and after (bottom) quality control. (C) UMAP visualization showing different tissue origins, with colours representing different tissue sources. (D) UMAP visualization displaying samples from different patients, with colours representing individual patients. (E) UMAP visualization showing the expression of canonical marker genes for major cell types. HE, haematoxylin–eosin; UMAP, Uniform Manifold Approximation and Projection.

**Figure S2:** Epithelial heterogeneity and functional states associated with LVSI in EEC. (A) Heatmap displaying representative genes for each epithelial subset. (B) Bubble plot showing GO‐enriched signalling pathways across tumour epithelial subsets. (C) Bubble plot displaying inflammatory, immune, and progesterone resistance‐related programs across tumour epithelial subsets. (D) Heatmap showing inflammatory and immune‐related states across tumour epithelial subsets. (E) Heatmap illustrating correlations among gene signature scores in tumour epithelial cells. (F) Differentially enriched pathways between malignant epithelial cells from LVSI+ and LVSI− EEC. LVSI, lymphovascular space invasion; EEC, endometrioid endometrial carcinoma; GO, Gene Ontology; LVSI+, LVSI present; LVSI−, LVSI absent; S, synthesis phase; G2/M, gap 2/mitosis phase; OXPHOS, oxidative phosphorylation; ER Stress, Endoplasmic Reticulum Stress; p‐EMT, partial epithelial–mesenchymal transition; EMT, epithelial–mesenchymal transition; OR, odds ratio.

**Figure S3:** T/NK‐cell heterogeneity and functional programs in LVSI+ and LVSI− EEC. (A) UMAP visualization showing the expression of canonical marker genes in T/NK cells. (B) UMAP visualization showing the expression of representative exhaustion‐related genes in T/NK cells. (C) Proportions of T/NK subsets in Normal, LVSI−, LVSI‐local and LVSI‐substantial samples, with colours indicating odds ratios. (D) Pathways upregulated in Cycling_CD8+ T cells compared with other T/NK subsets. (E) Pathways downregulated in Cycling_CD8+ T cells compared with other T/NK subsets. (F) Pathway enrichment analysis of differentially expressed genes in ZNF683+ SOX4+ CD8+ T cells compared with Cycling_CD8+ T cells. (G) Pseudotime trajectory of CD4+ T‐cell differentiation. (H) Heatmap showing key genes driving branch decisions along the CD4+ T‐cell trajectory. T/NK, T cell and NK cell; LVSI, lymphovascular space invasion; LVSI+, LVSI present; LVSI−, LVSI absent; EEC, endometrioid endometrial carcinoma; UMAP, Uniform Manifold Approximation and Projection.

**Figure S4:** Monocyte–macrophage and dendritic cell heterogeneity in LVSI+ and LVSI− EEC. (A) UMAP visualization of signature scores across monocyte–macrophage subsets. (B) Pathway enrichment analysis of the SPP1 + MMP9 + Mac subset. (C) UMAP visualization of dendritic cell subsets, with colours representing distinct subsets. (D) Dot plot showing marker gene expression across dendritic cell subsets, with colour indicating normalized expression level and dot size representing the proportion of expressing cells. (E) Proportions of dendritic cell subsets in Normal, LVSI−, LVSI‐local and LVSI‐substantial samples, with colours indicating odds ratios. (F) Correlation between the proportions of cDC2_LAMP3_DC and pDC subsets. (G) Dot plot showing the expression of signature genes across dendritic cell subsets. (H) Expression of MHC class I and class II molecules across dendritic cell subsets. (I) Activation, migration, S‐phase, and G2M‐phase scores across dendritic cell subsets. (J) Violin plots showing the expression of representative immunosuppressive genes in Normal, LVSI−, and LVSI+ samples. (K) Pathways significantly upregulated in the LAMP3_DC subset compared with other DC subsets. (L) Bar plot showing pathways enriched in cDC1_LAMP3_DC and cDC2_LAMP3_DC subsets. (M) Violin plots showing the expression of key genes along the LAMP3_DC trajectory. (N) Differentially enriched pathways in the LAMP3_DC subset between LVSI+ and LVSI− EEC. LVSI, lymphovascular space invasion; LVSI+, LVSI present; LVSI−, LVSI absent; EEC, endometrioid endometrial carcinoma; UMAP, Uniform Manifold Approximation and Projection; TAM, tumour‐associated macrophage; MHC, major histocompatibility complex.

**Figure S5:** LVSI‐associated ligand expression and signalling interactions in EEC. (A) Heatmap showing the expression of LVSI+‐specific ligands across cell types. (B) Lollipop plot showing differences in interaction probabilities between LVSI+ and LVSI− cell subsets. (C) Heatmap showing interaction probabilities from SPP1 + MMP9 + Mac and WNT5A_mCAF fibroblasts to endothelial (left) and T/NK (right) subsets. LVSI, lymphovascular space invasion; LVSI+, LVSI present; LVSI−, LVSI absent; EEC, endometrioid endometrial carcinoma; T/NK, T cell and NK cell.

**Figure S6:** Differential ligand–receptor interactions between LVSI+ and LVSI− EEC‐derived cells. (A) Bubble plot showing interactions from TC4 to other cell subsets in LVSI+ and LVSI− EEC. Statistically significant ligand–receptor pairs (*p* < 0.05) are shown. Text colour represents tissue origin, and dot colour indicates interaction probability. (B) Bubble plot showing interactions from WNT5A_mCAF or SPP1 + MMP9 + Mac to LECs or Tip_ECs in LVSI+ and LVSI− EEC. Statistically significant ligand–receptor pairs (*p* < 0.05) are shown. Text colour represents tissue origin, and dot colour indicates interaction probability. (C) Bubble plot showing interactions from WNT5A_mCAF or SPP1 + MMP9 + Mac to T/NK subsets in LVSI+ and LVSI− EEC. Statistically significant ligand–receptor pairs (*p* < 0.05) are shown. Text colour represents tissue origin, and dot colour indicates interaction probability. (D) Bubble plot showing interactions from LECs or Tip_ECs to T/NK subsets in LVSI+ and LVSI− EEC. Statistically significant ligand–receptor pairs (*p* < 0.05) are shown. Text colour represents tissue origin, and dot colour indicates interaction probability. LVSI, lymphovascular space invasion; LVSI+, LVSI present; LVSI−, LVSI absent; EEC, endometrioid endometrial carcinoma; T/NK, T cell and NK cell.

**Figure S7:** Spatial features of invasive epithelial cells across LVSI stages in EEC. Representative HE and mIF images showing the spatial distribution of Ki67, HIF1A, EPCAM, and CD44 across LVSI−, LVSI‐local, and LVSI‐substantial EEC. Solid lines denote the epithelial–stromal boundary, and dashed circles indicate regions with high expression. Black arrows (HE) and short white arrows (mIF) mark the locations of the magnified areas shown on the right. Long white arrows indicate the direction of tumour invasion. Nuclei are counterstained with DAPI. Scale bars are indicated. LVSI, lymphovascular space invasion; EEC, endometrioid endometrial carcinoma; HE, haematoxylin–eosin; mIF, multiplex immunofluorescence.

**Figure S8:** Spatial distribution of the immune microenvironment across LVSI stages in EEC. Representative mIF images showing the spatial distribution and colocalization of CD68, PD‐L1, EPCAM, and CD3 across LVSI−, LVSI‐local, and LVSI‐substantial EEC. PD‐L1+ macrophages are shown from tumour stroma and glandular lumen to peritumoral stroma and invasive tumour clusters. Solid circles and short white arrows indicate the magnified regions shown on the right. Dashed circles in the upper‐left first row mark regions with enriched CD3 expression, and in the second row mark regions with enriched CD68 expression. Nuclei are counterstained with DAPI. Scale bars are indicated. LVSI, lymphovascular space invasion; EEC, endometrioid endometrial carcinoma; mIF, multiplex immunofluorescence; PD‐L1, programmed death‐ligand 1.

**Figure S9:** Spatial distribution and phenotypic features of T cells across LVSI stages in EEC. Representative mIF images showing the spatial distribution and colocalization of Ki67, PD‐1, CD8, and CD4 across LVSI−, LVSI‐local, and LVSI‐substantial EEC. Images depict T‐cell localization from the tumour stroma and basal regions to the peritumoral stroma. Solid boxes on the left indicate the regions magnified on the right. Short white arrows denote cells co‐expressing Ki67 or PD‐1 with CD8 or CD4. Cyan short arrows indicate cells co‐expressing Ki67, PD‐1, and CD8, and dark yellow short arrows indicate cells co‐expressing Ki67, PD‐1, and CD4. Nuclei are counterstained with DAPI. Scale bars are indicated. LVSI, lymphovascular space invasion; EEC, endometrioid endometrial carcinoma; mIF, multiplex immunofluorescence; PD‐1, programmed cell death protein 1.

**Figure S10:** Schematic overview of cellular crosstalk within the metastatic tumour microenvironment during LVSI in EEC. Schematic illustration summarizing dynamic interactions among malignant epithelial cells, immune cells, and stromal cells during LVSI progression, integrating single‐cell RNA sequencing, ligand–receptor characterization, and spatial information. Potential therapeutic targets are indicated. LVSI, lymphovascular space invasion; EEC, endometrioid endometrial carcinoma; scRNA‐seq, single‐cell RNA sequencing.

**Table S1:** Functional annotation of nine LVSI‐associated prognostic genes.

**Table S2:** Characteristics of the single‐cell sequencing cohort across different LVSI statuses in EEC.

**Table S3:** Characteristics of multiplex immunofluorescence across different LVSI statuses in EEC.

## Data Availability

The raw sequence data are deposited in the Genome Sequence Archive [81] at National Genomics Data Center [82], China National Center for Bioinformation/Beijing Institute of Genomics, Chinese Academy of Sciences (GSA‐Human: HRA015421), publicly accessible at https://ngdc.cncb.ac.cn/gsa‐human.
