# Supplementary figures and images for: A Multicellular Coordinated Network Driving Lymphovascular Space Invasion in Endometrioid Endometrial Carcinoma
Source: Cell Prolif. 2026 Jun 23:e70246. Online ahead of print. doi: 10.1111/cpr.70246 (PMC13325826; doi:10.1111/cpr.70246)

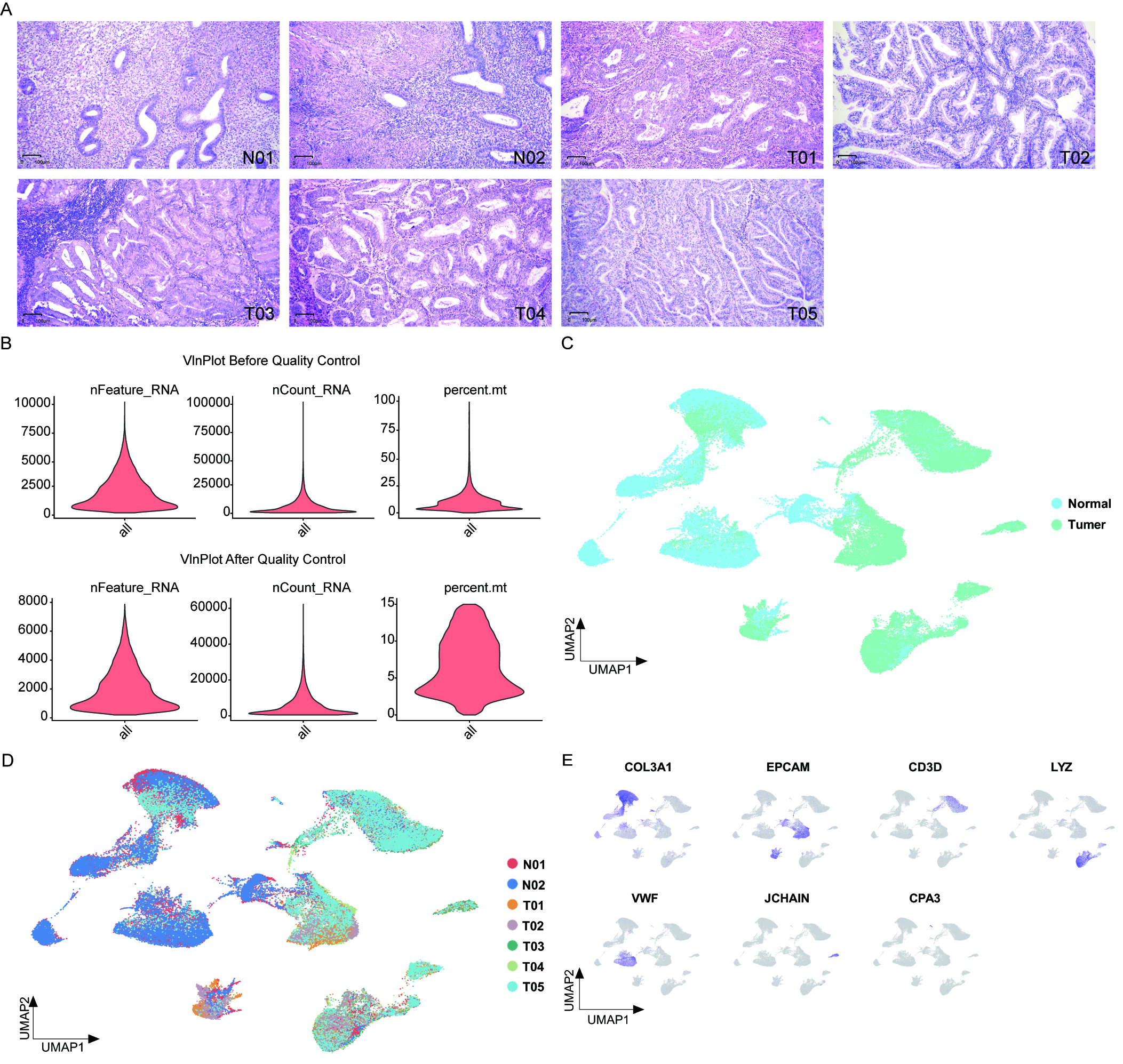

Supplement: Supplementary file 1 — Figure S1: Data quality control and filtering. (A) HE staining of samples derived from seven patients. (B) Violin plots illustrating nFeature_RNA, nCount_RNA, and mitochondrial gene expression (percent mt) before (top) and after (bottom) quality control. (C) UMAP visualization showing different tissue origins, with colours representing different tissue sources. (D) UMAP visualization displaying samples from different patients, with colours representing individual patients. (E) UMAP visualization showing the expression of canonical marker genes for major cell types. HE, haematoxylin–eosin; UMAP, Uniform Manifold Approximation and Projection. [file CPR-9999-e70246-s001.tif]

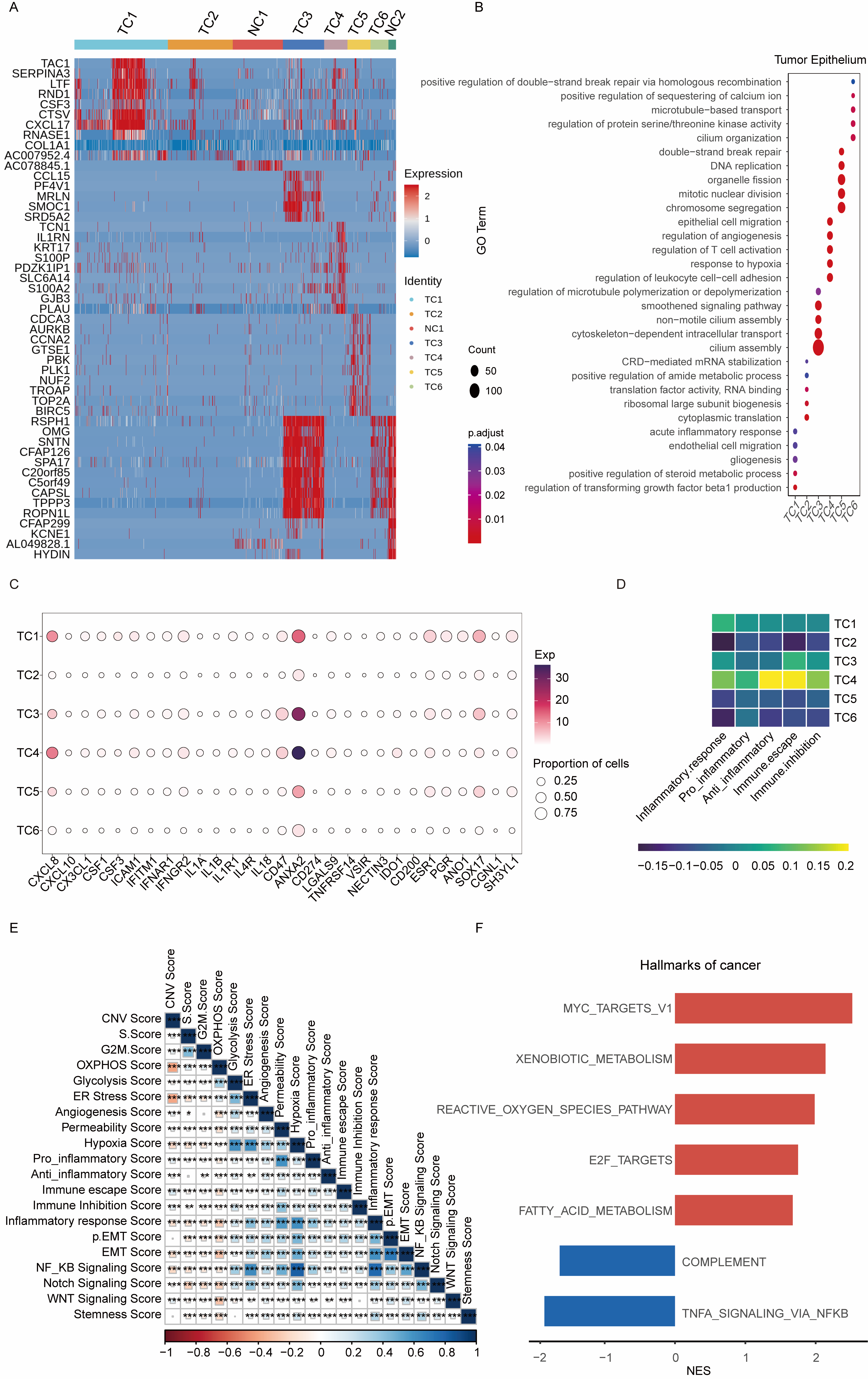

Supplement: Supplementary file 2 — Figure S2: Epithelial heterogeneity and functional states associated with LVSI in EEC. (A) Heatmap displaying representative genes for each epithelial subset. (B) Bubble plot showing GO‐enriched signalling pathways across tumour epithelial subsets. (C) Bubble plot displaying inflammatory, immune, and progesterone resistance‐related programs across tumour epithelial subsets. (D) Heatmap showing inflammatory and immune‐related states across tumour epithelial subsets. (E) Heatmap illustrating correlations among gene signature scores in tumour epithelial cells. (F) Differentially enriched pathways between malignant epithelial cells from LVSI+ and LVSI− EEC. LVSI, lymphovascular space invasion; EEC, endometrioid endometrial carcinoma; GO, Gene Ontology; LVSI+, LVSI present; LVSI−, LVSI absent; S, synthesis phase; G2/M, gap 2/mitosis phase; OXPHOS, oxidative phosphorylation; ER Stress, Endoplasmic Reticulum Stress; p‐EMT, partial epithelial–mesenchymal transition; EMT, epithelial–mesenchymal transition; OR, odds ratio. [file CPR-9999-e70246-s002.tif]

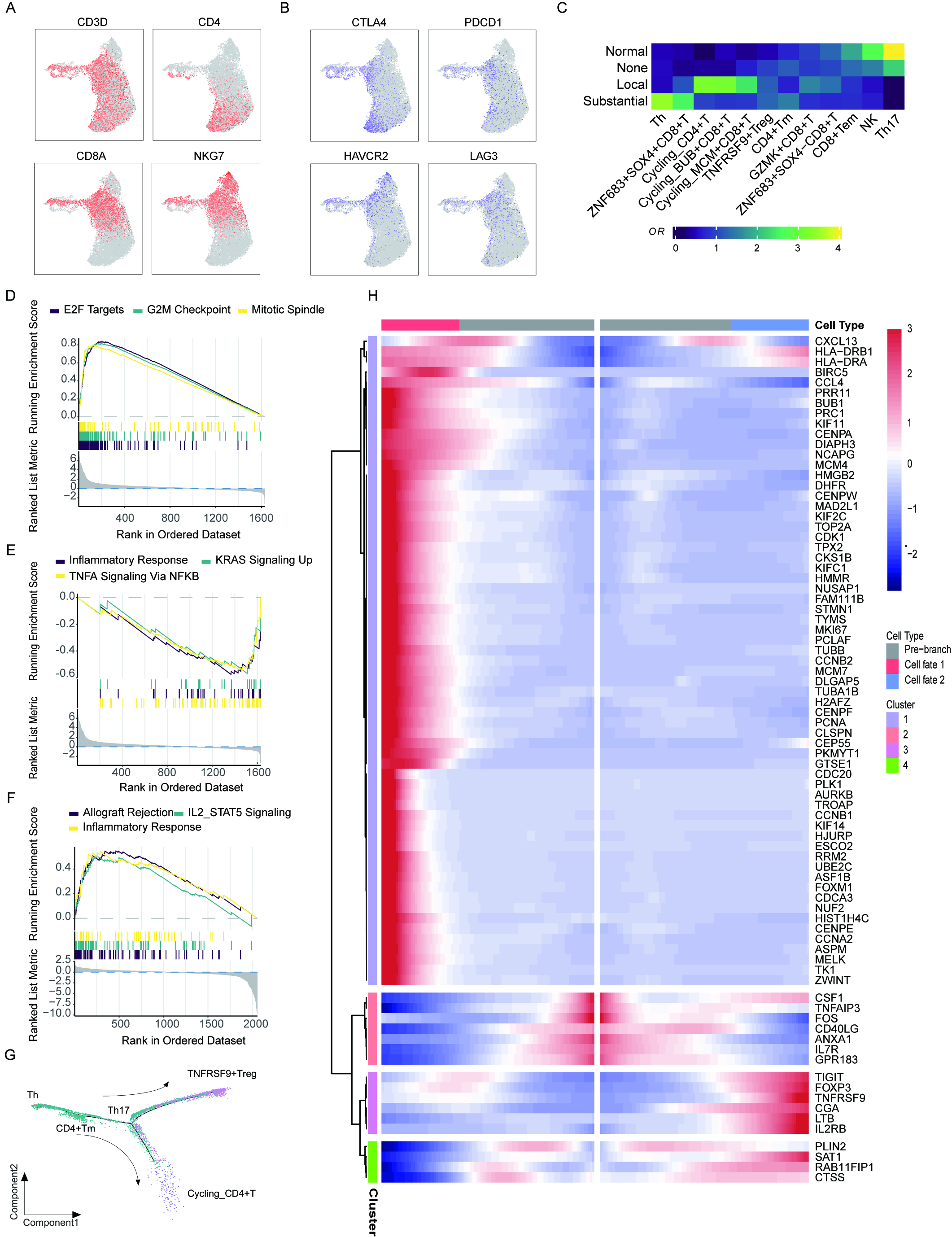

Supplement: Supplementary file 3 — Figure S3: T/NK‐cell heterogeneity and functional programs in LVSI+ and LVSI− EEC. (A) UMAP visualization showing the expression of canonical marker genes in T/NK cells. (B) UMAP visualization showing the expression of representative exhaustion‐related genes in T/NK cells. (C) Proportions of T/NK subsets in Normal, LVSI−, LVSI‐local and LVSI‐substantial samples, with colours indicating odds ratios. (D) Pathways upregulated in Cycling_CD8+ T cells compared with other T/NK subsets. (E) Pathways downregulated in Cycling_CD8+ T cells compared with other T/NK subsets. (F) Pathway enrichment analysis of differentially expressed genes in ZNF683+ SOX4+ CD8+ T cells compared with Cycling_CD8+ T cells. (G) Pseudotime trajectory of CD4+ T‐cell differentiation. (H) Heatmap showing key genes driving branch decisions along the CD4+ T‐cell trajectory. T/NK, T cell and NK cell; LVSI, lymphovascular space invasion; LVSI+, LVSI present; LVSI−, LVSI absent; EEC, endometrioid endometrial carcinoma; UMAP, Uniform Manifold Approximation and Projection. [file CPR-9999-e70246-s010.tif]

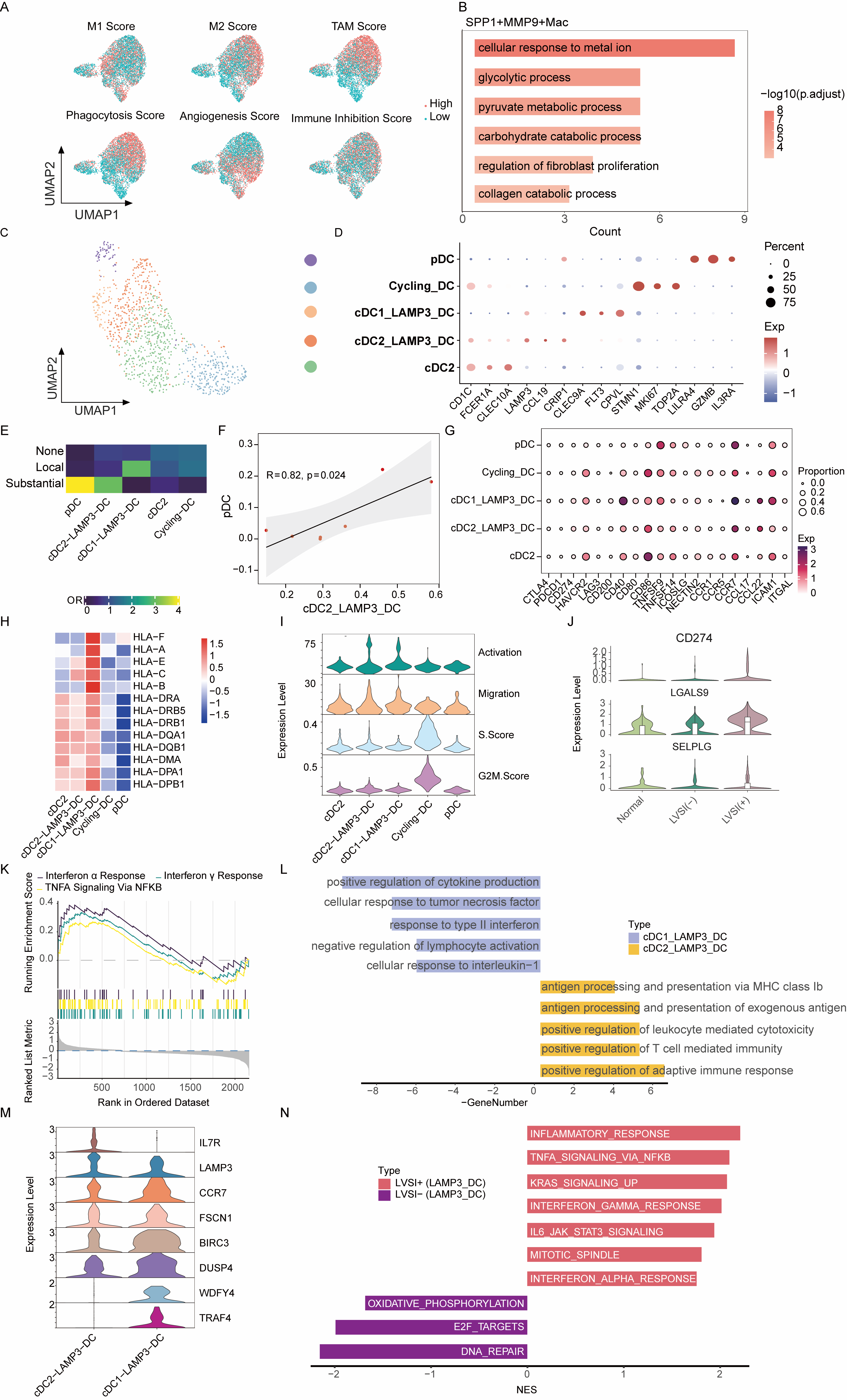

Supplement: Supplementary file 4 — Figure S4: Monocyte–macrophage and dendritic cell heterogeneity in LVSI+ and LVSI− EEC. (A) UMAP visualization of signature scores across monocyte–macrophage subsets. (B) Pathway enrichment analysis of the SPP1 + MMP9 + Mac subset. (C) UMAP visualization of dendritic cell subsets, with colours representing distinct subsets. (D) Dot plot showing marker gene expression across dendritic cell subsets, with colour indicating normalized expression level and dot size representing the proportion of expressing cells. (E) Proportions of dendritic cell subsets in Normal, LVSI−, LVSI‐local and LVSI‐substantial samples, with colours indicating odds ratios. (F) Correlation between the proportions of cDC2_LAMP3_DC and pDC subsets. (G) Dot plot showing the expression of signature genes across dendritic cell subsets. (H) Expression of MHC class I and class II molecules across dendritic cell subsets. (I) Activation, migration, S‐phase, and G2M‐phase scores across dendritic cell subsets. (J) Violin plots showing the expression of representative immunosuppressive genes in Normal, LVSI−, and LVSI+ samples. (K) Pathways significantly upregulated in the LAMP3_DC subset compared with other DC subsets. (L) Bar plot showing pathways enriched in cDC1_LAMP3_DC and cDC2_LAMP3_DC subsets. (M) Violin plots showing the expression of key genes along the LAMP3_DC trajectory. (N) Differentially enriched pathways in the LAMP3_DC subset between LVSI+ and LVSI− EEC. LVSI, lymphovascular space invasion; LVSI+, LVSI present; LVSI−, LVSI absent; EEC, endometrioid endometrial carcinoma; UMAP, Uniform Manifold Approximation and Projection; TAM, tumour‐associated macrophage; MHC, major histocompatibility complex. [file CPR-9999-e70246-s007.tif]

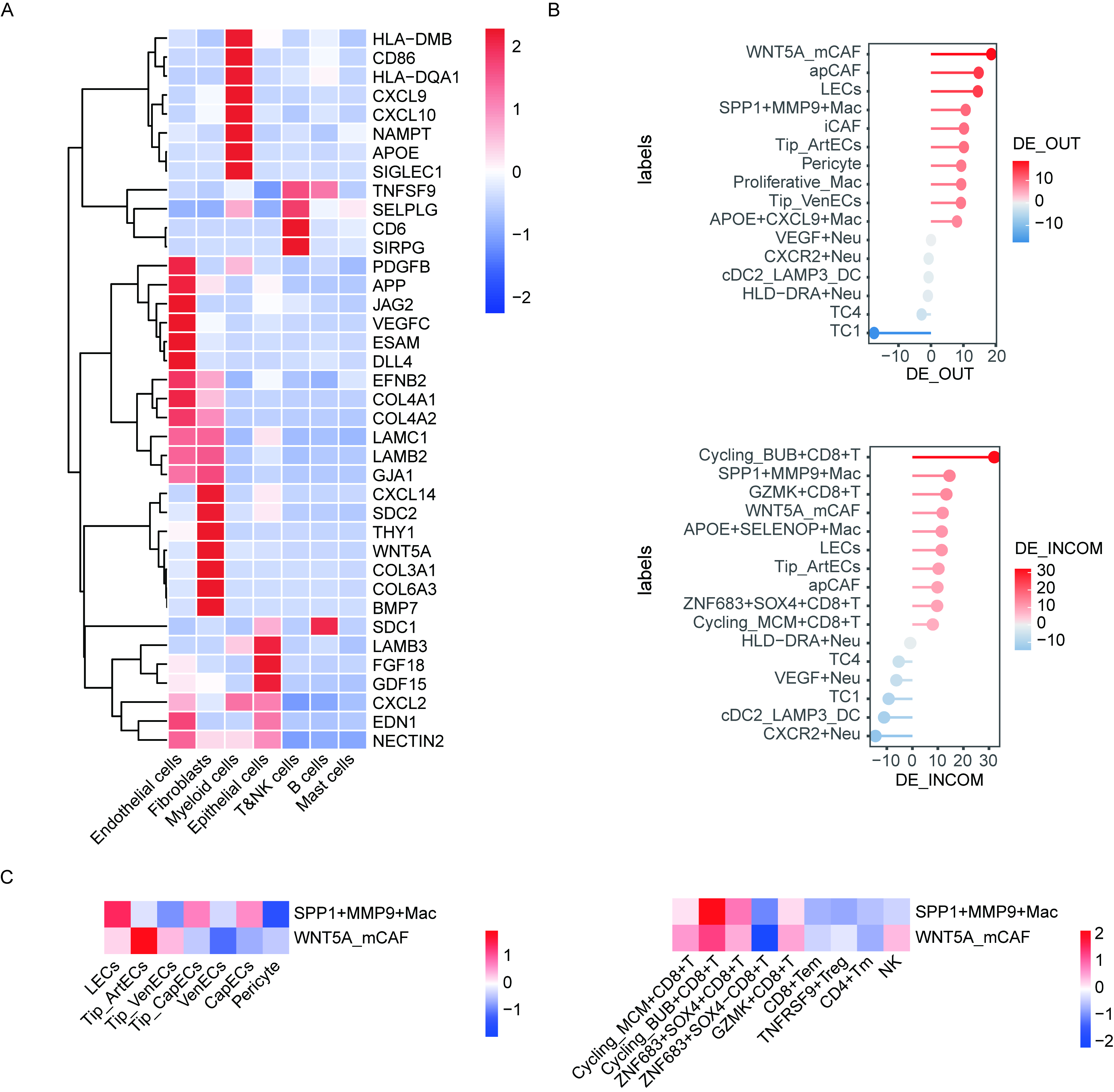

Supplement: Supplementary file 5 — Figure S5: LVSI‐associated ligand expression and signalling interactions in EEC. (A) Heatmap showing the expression of LVSI+‐specific ligands across cell types. (B) Lollipop plot showing differences in interaction probabilities between LVSI+ and LVSI− cell subsets. (C) Heatmap showing interaction probabilities from SPP1 + MMP9 + Mac and WNT5A_mCAF fibroblasts to endothelial (left) and T/NK (right) subsets. LVSI, lymphovascular space invasion; LVSI+, LVSI present; LVSI−, LVSI absent; EEC, endometrioid endometrial carcinoma; T/NK, T cell and NK cell. [file CPR-9999-e70246-s012.tif]

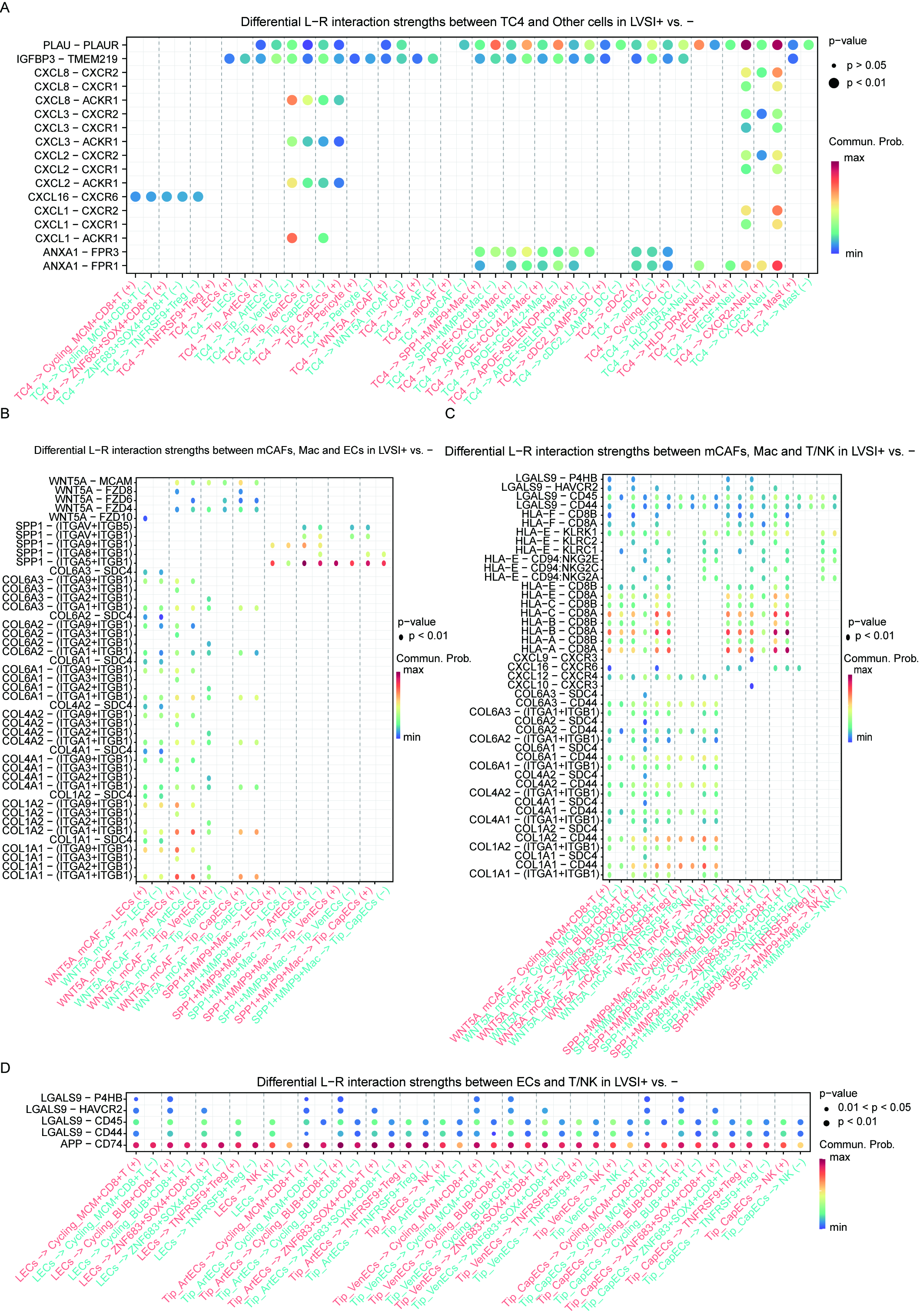

Supplement: Supplementary file 6 — Figure S6: Differential ligand–receptor interactions between LVSI+ and LVSI− EEC‐derived cells. (A) Bubble plot showing interactions from TC4 to other cell subsets in LVSI+ and LVSI− EEC. Statistically significant ligand–receptor pairs (p < 0.05) are shown. Text colour represents tissue origin, and dot colour indicates interaction probability. (B) Bubble plot showing interactions from WNT5A_mCAF or SPP1 + MMP9 + Mac to LECs or Tip_ECs in LVSI+ and LVSI− EEC. Statistically significant ligand–receptor pairs (p < 0.05) are shown. Text colour represents tissue origin, and dot colour indicates interaction probability. (C) Bubble plot showing interactions from WNT5A_mCAF or SPP1 + MMP9 + Mac to T/NK subsets in LVSI+ and LVSI− EEC. Statistically significant ligand–receptor pairs (p < 0.05) are shown. Text colour represents tissue origin, and dot colour indicates interaction probability. (D) Bubble plot showing interactions from LECs or Tip_ECs to T/NK subsets in LVSI+ and LVSI− EEC. Statistically significant ligand–receptor pairs (p < 0.05) are shown. Text colour represents tissue origin, and dot colour indicates interaction probability. LVSI, lymphovascular space invasion; LVSI+, LVSI present; LVSI−, LVSI absent; EEC, endometrioid endometrial carcinoma; T/NK, T cell and NK cell. [file CPR-9999-e70246-s003.tif]

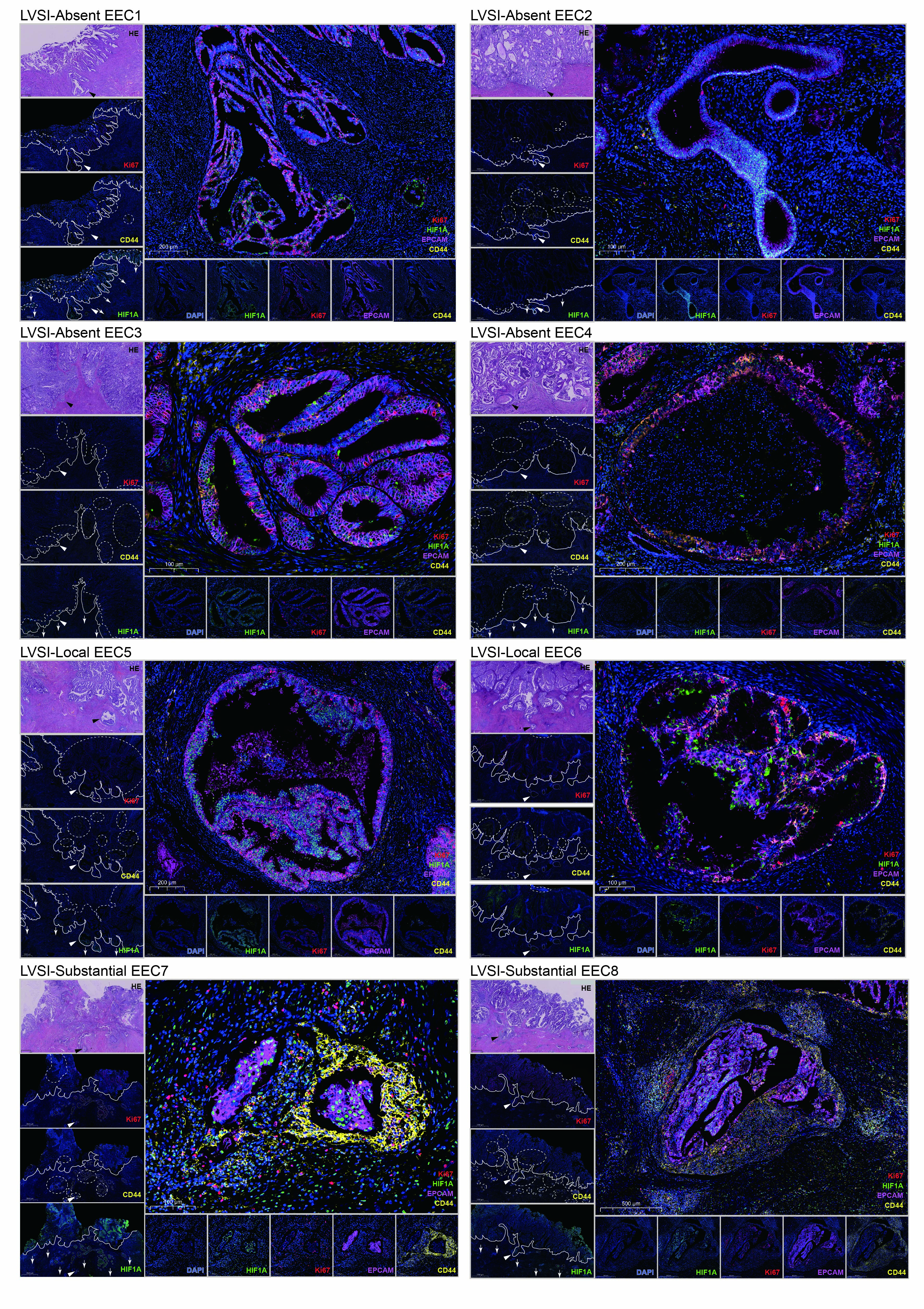

Supplement: Supplementary file 7 — Figure S7: Spatial features of invasive epithelial cells across LVSI stages in EEC. Representative HE and mIF images showing the spatial distribution of Ki67, HIF1A, EPCAM, and CD44 across LVSI−, LVSI‐local, and LVSI‐substantial EEC. Solid lines denote the epithelial–stromal boundary, and dashed circles indicate regions with high expression. Black arrows (HE) and short white arrows (mIF) mark the locations of the magnified areas shown on the right. Long white arrows indicate the direction of tumour invasion. Nuclei are counterstained with DAPI. Scale bars are indicated. LVSI, lymphovascular space invasion; EEC, endometrioid endometrial carcinoma; HE, haematoxylin–eosin; mIF, multiplex immunofluorescence. [file CPR-9999-e70246-s011.jpg]

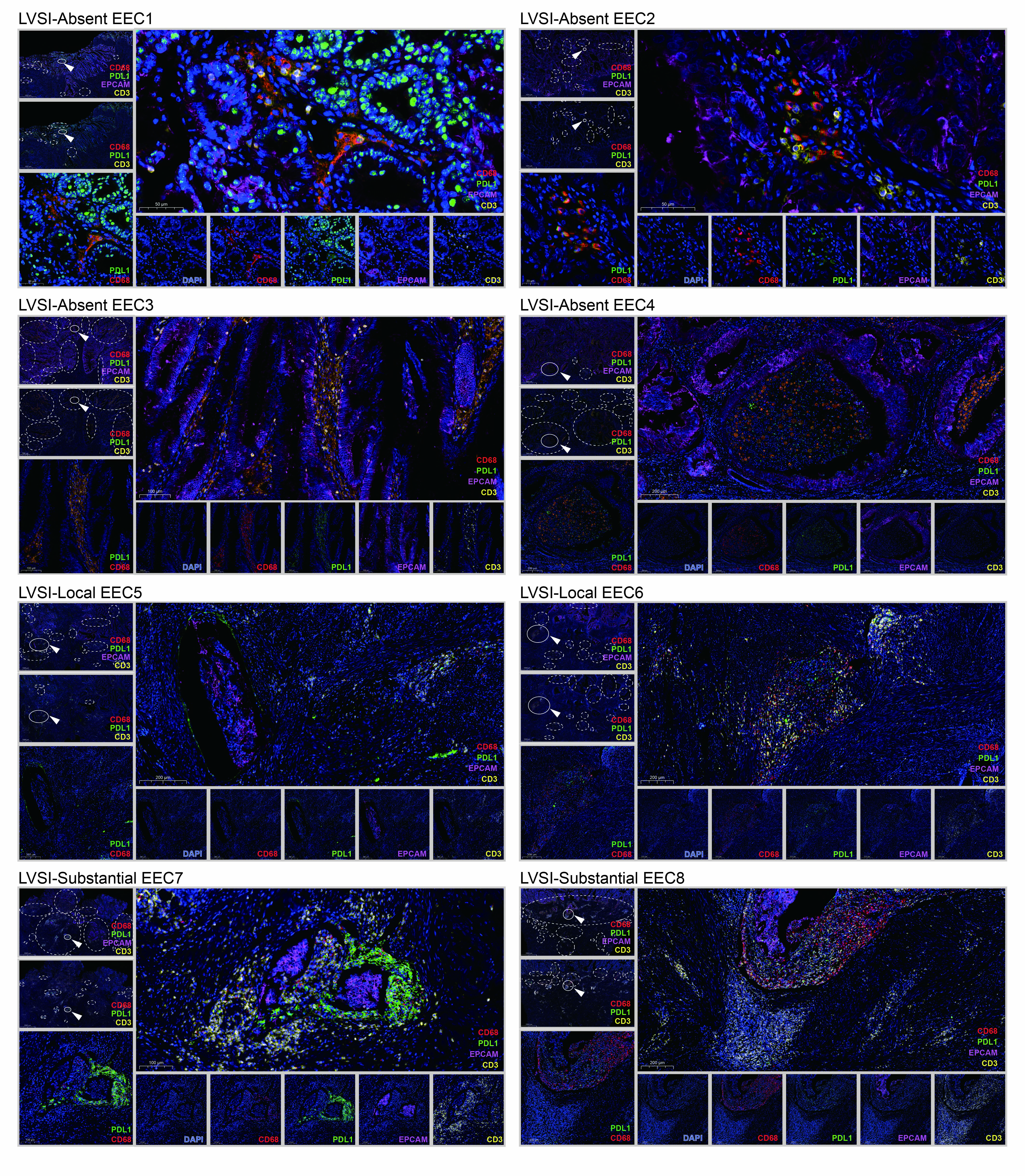

Supplement: Supplementary file 8 — Figure S8: Spatial distribution of the immune microenvironment across LVSI stages in EEC. Representative mIF images showing the spatial distribution and colocalization of CD68, PD‐L1, EPCAM, and CD3 across LVSI−, LVSI‐local, and LVSI‐substantial EEC. PD‐L1+ macrophages are shown from tumour stroma and glandular lumen to peritumoral stroma and invasive tumour clusters. Solid circles and short white arrows indicate the magnified regions shown on the right. Dashed circles in the upper‐left first row mark regions with enriched CD3 expression, and in the second row mark regions with enriched CD68 expression. Nuclei are counterstained with DAPI. Scale bars are indicated. LVSI, lymphovascular space invasion; EEC, endometrioid endometrial carcinoma; mIF, multiplex immunofluorescence; PD‐L1, programmed death‐ligand 1. [file CPR-9999-e70246-s006.jpg]

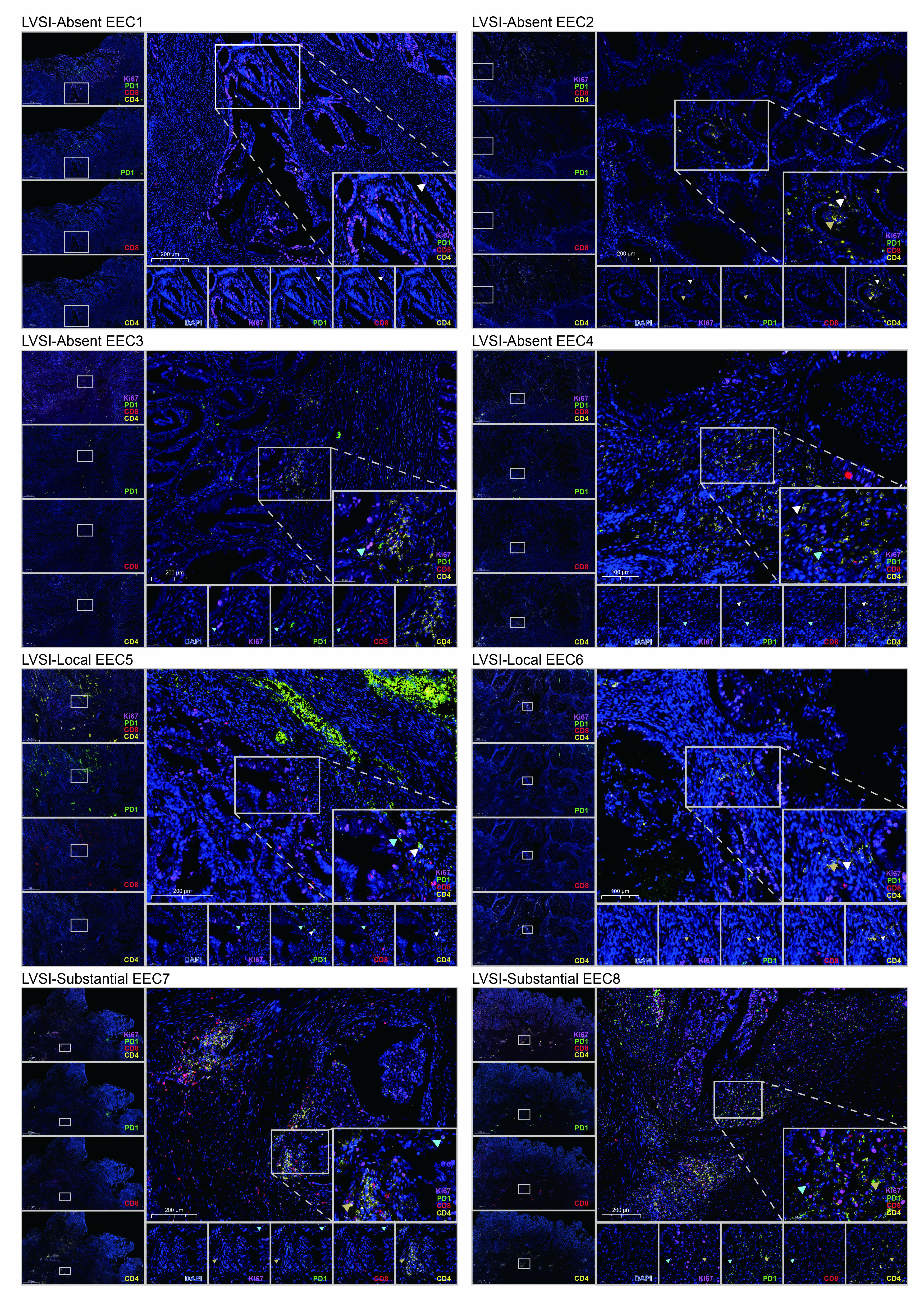

Supplement: Supplementary file 9 — Figure S9: Spatial distribution and phenotypic features of T cells across LVSI stages in EEC. Representative mIF images showing the spatial distribution and colocalization of Ki67, PD‐1, CD8, and CD4 across LVSI−, LVSI‐local, and LVSI‐substantial EEC. Images depict T‐cell localization from the tumour stroma and basal regions to the peritumoral stroma. Solid boxes on the left indicate the regions magnified on the right. Short white arrows denote cells co‐expressing Ki67 or PD‐1 with CD8 or CD4. Cyan short arrows indicate cells co‐expressing Ki67, PD‐1, and CD8, and dark yellow short arrows indicate cells co‐expressing Ki67, PD‐1, and CD4. Nuclei are counterstained with DAPI. Scale bars are indicated. LVSI, lymphovascular space invasion; EEC, endometrioid endometrial carcinoma; mIF, multiplex immunofluorescence; PD‐1, programmed cell death protein 1. [file CPR-9999-e70246-s013.jpg]

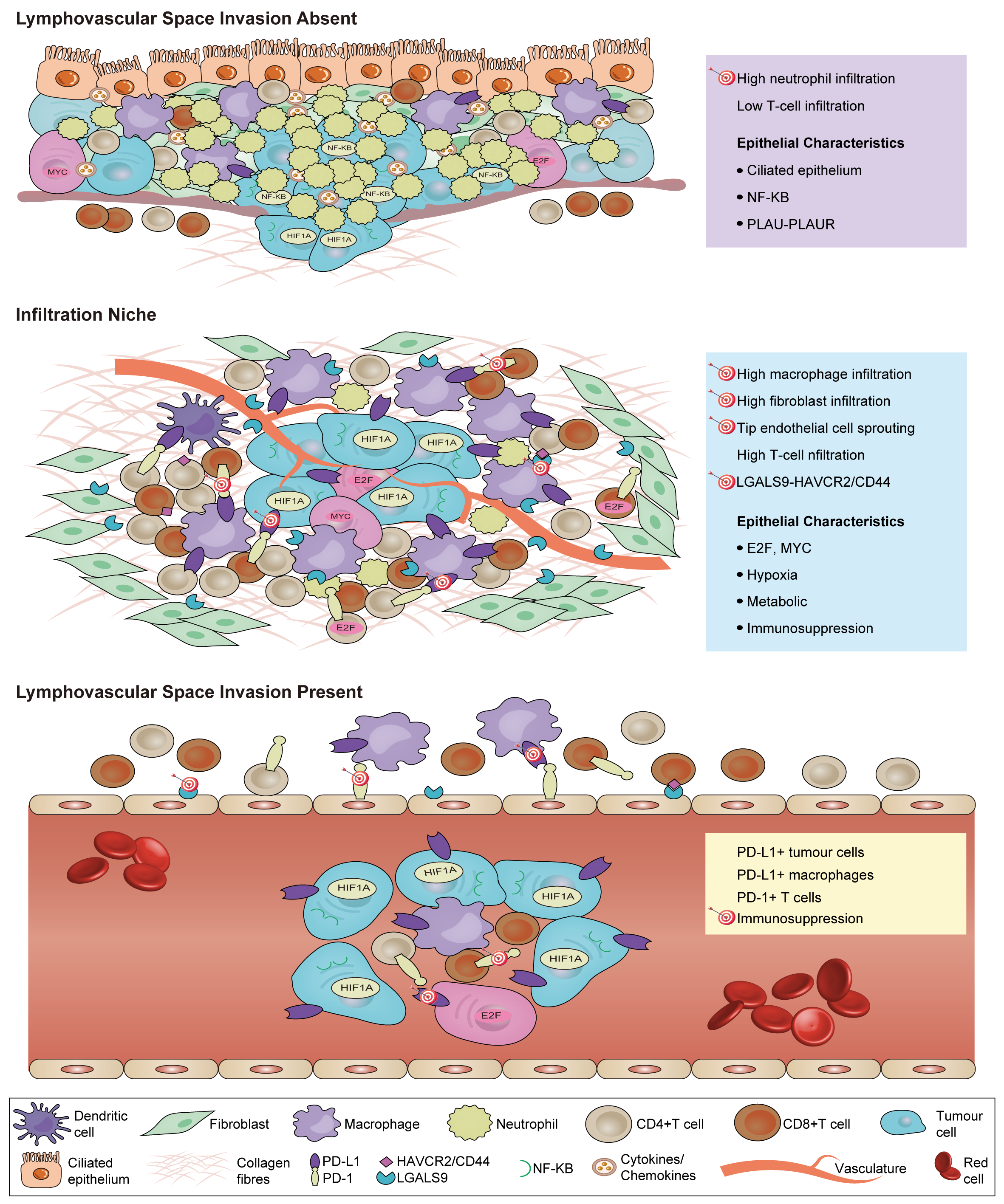

Supplement: Supplementary file 10 — Figure S10: Schematic overview of cellular crosstalk within the metastatic tumour microenvironment during LVSI in EEC. Schematic illustration summarizing dynamic interactions among malignant epithelial cells, immune cells, and stromal cells during LVSI progression, integrating single‐cell RNA sequencing, ligand–receptor characterization, and spatial information. Potential therapeutic targets are indicated. LVSI, lymphovascular space invasion; EEC, endometrioid endometrial carcinoma; scRNA‐seq, single‐cell RNA sequencing. [file CPR-9999-e70246-s009.tif]
